# Maternal Vitamin C Intake during Pregnancy Influences Long-Term Offspring Growth with Timing- and Sex-Specific Effects in Guinea Pigs

**DOI:** 10.3390/nu16030369

**Published:** 2024-01-26

**Authors:** Sharna J. Coker, Mary J. Berry, Margreet C. M. Vissers, Rebecca M. Dyson

**Affiliations:** 1 Perinatal and Developmental Physiology Group, Department of Paediatrics and Child Health, University of Otago, Wellington 6242, New Zealand; max.berry@otago.ac.nz (M.J.B.); becs.dyson@otago.ac.nz (R.M.D.); 2 Mātai Hāora-Centre for Redox Biology and Medicine, Department of Pathology and Biomedical Science, University of Otago, Christchurch 8140, New Zealand; margreet.vissers@otago.ac.nz

**Keywords:** vitamin C (ascorbate; ascorbic acid), pregnancy, maternal diet, foetal programming, foetal growth, postnatal growth, metabolic function, guinea pig

## Abstract

Our previous work in guinea pigs revealed that low vitamin C intake during preconception and pregnancy adversely affects fertility, pregnancy outcomes, and foetal and neonatal growth in a sex-dependent manner. To investigate the long-term impact on offspring, we monitored their growth from birth to adolescence (four months), recorded organ weights at childhood equivalence (28 days) and adolescence, and assessed physiological parameters like oral glucose tolerance and basal cortisol concentrations. We also investigated the effects of the timing of maternal vitamin C restriction (early vs. late gestation) on pregnancy outcomes and the health consequences for offspring. Dunkin Hartley guinea pigs were fed an optimal (900 mg/kg feed) or low (100 mg/kg feed) vitamin C diet ad libitum during preconception. Pregnant dams were then randomised into four feeding regimens: consistently optimal, consistently low, low during early pregnancy, or low during late pregnancy. We found that low maternal vitamin C intake during early pregnancy accelerated foetal and neonatal growth in female offspring and altered glucose homeostasis in the offspring of both sexes at an age equivalent to early childhood. Conversely, low maternal vitamin C intake during late pregnancy resulted in foetal growth restriction and reduced weight gain in male offspring throughout their lifespan. We conclude that altered vitamin C during development has long-lasting, sex-specific consequences for offspring and that the timing of vitamin C depletion is also critical, with low levels during early development being associated with the development of a metabolic syndrome-related phenotype, while later deprivation appears to be linked to a growth-faltering phenotype.

## 1. Introduction

There is now compelling evidence from experimental, clinical, and epidemiological studies that maternal malnutrition during pregnancy can have permanent effects on offspring’s organ structure and function and predispose them to developing chronic conditions later in life. These conditions are commonly associated with metabolic dysfunction, and include obesity, diabetes, hypertension, and cardiovascular disease [1,2,3,4,5,6]. Research has also shown that the timing during gestation at which the nutritional insult occurs is critical in determining the future disease risk of the offspring [7,8].

Vitamin C (ascorbate; ascorbic acid) is an essential nutrient that is sourced exclusively from the diet in humans and a few other species, including guinea pigs, due to a non-functioning gulonolactone oxidase gene [9]. Human and guinea pig foetuses access vitamin C via sodium-dependent vitamin C (SVCT) transporters in the placenta and therefore rely on adequate maternal intake throughout pregnancy [10,11]. In humans, low maternal vitamin C status has been associated with low-birth-weight infants [12,13] and poor infant growth up to six months of age [14]. Animal studies have revealed the severe consequences that can arise for offspring when the mother experiences extreme vitamin C deficiency, including growth, cardiovascular, pulmonary, hepatic, and vertebral defects [15,16,17], impaired brain development [18,19,20,21], and embryonic or foetal death [11,22]. Research on the effects of moderate vitamin C deficiency or suboptimal intake during pregnancy and the long-term consequences for offspring remains limited.

Programming of the hypothalamic–pituitary–adrenal (HPA) axis begins in utero and increased activity just before birth is normal for the maturation of the lungs and surfactant supply [23]. In animal models, various prenatal challenges such as malnutrition [24], stress exposure [25,26], and preterm birth [27] have been shown to cause disturbances in HPA axis function that persist in later life. Clinically, dysregulation of HPA axis activity has been associated with a wide variety of physiological disorders including diabetes [28], hypertension [29], and neurological pathologies such as depression, anxiety, schizophrenia, and others [30,31,32,33]. Both in humans and our guinea pig model, the highest concentrations of vitamin C are found in the brain and the adrenal glands [34,35,36]. Therefore, we hypothesised that the developing HPA axis would be particularly vulnerable to vitamin C depletion in utero and may be important in regulating foetal adaptive metabolic responses.

Our previous work examined the impact of consistently low vitamin C status on maternal and neonatal characteristics as well as pregnancy outcomes in guinea pigs [36]. We have shown that low vitamin C intake adversely affects fertility, pregnancy outcomes, and foetal and neonatal growth. To better understand offspring predisposition to later-life disease following reduced vitamin C availability in utero, the current study aimed to characterise the phenotypic effects of low maternal vitamin C consumption upon offspring physiology and metabolic status across the lifespan. Furthermore, we aimed to identify specific developmental timepoints that are sensitive to vitamin C depletion by examining outcomes for pregnant dams and their offspring that were exposed to varying levels of vitamin C during different stages of pregnancy, specifically comparing low vitamin C intake during early vs. late pregnancy.

We report that maternal vitamin C intake has significant timing- and sex-specific effects on the long-term growth and metabolic function of offspring. The potential biological mechanisms and the implications for translating these findings to humans is discussed.

## 2. Materials and Methods

### 2.1. Ethics

This research received prospective approval from the Animal Ethics Committee of the University of Otago Wellington (AEC: 20–85). All protocols adhered to the Health Research Council of New Zealand code of practice for the ethical treatment and utilization of animals in scientific research. Data are presented in accordance with the ARRIVE (Animals in Research: Reporting In Vivo Experiments) guidelines [37].

### 2.2. Animal Model

Animals for this study were obtained from the outbred colony of Dunkin Hartley guinea pigs maintained in the Biomedical Research Unit at the University of Otago Wellington and the Small Animal Production Unit at Massey University, Palmerston North. Female guinea pigs were accommodated in group housing within floor pens, and male guinea pigs were individually housed in cages. The animals were kept under a 12 h day/night light cycle, and the facilities maintained a controlled environment at 20–23 °C and 50–70% humidity.

At eight weeks of age, 117 virgin females were randomised into a total of four feeding regimens: optimal vitamin C intake (900 mg vitamin C/kg feed) for the duration of pregnancy (optimal, *n* = 35), low vitamin C intake (100 mg vitamin C/kg feed) for the duration of pregnancy (low, *n* = 38), low vitamin C intake for the first trimester followed by optimal intake for the remainder of pregnancy (low-optimal, *n* = 22), and optimal vitamin C intake for the first trimester followed by low intake for the remainder of pregnancy (optimal-low, *n* = 22). The two feeds (Sharpes Feed Barn, Lower Hutt, New Zealand) were matched for all other macro- and micronutrients except vitamin C content. We have previously shown that a diet containing 100 mg vitamin C/kg of feed successfully induces a non-scorbutic vitamin C deficiency in guinea pigs of both sexes and of various adult ages, including during pregnancy [36]. Animals had unrestricted access to chow and drinking water. Their diets were supplemented with ~20 g of dried hay per animal daily, which was vitamin C-free after autoclaving at 120 °C. Throughout the study, animals were closely monitored for general welfare indicators twice daily and weighed weekly. As per our previously published breeding protocol [36], breeding commenced after a diet acclimation period lasting a minimum of three weeks. Dams were paired with a non-lineage and diet-matched sire for 72 h during oestrous to ensure timed mating. The date of conception/gestational age (GA) zero was defined as the midpoint of this mating period. Confirmation of pregnancy and determination of litter size (number of pups) were achieved through abdominal ultrasound, detectable around gestational day (GD) 21. On GD 24, which marks the end of the first trimester in guinea pigs, the dams allocated to the low-optimal and optimal-low groups swapped diets.

### 2.3. Delivery and Postnatal Care of Offspring

Dams gave birth spontaneously around gestational day (GD) 69, and the pups were randomly allocated to one of three weight-stratified groups: those euthanized within 24 h of birth (neonates), those euthanized at 28 days of age (juveniles), and those euthanized at four months of age (adolescents). The total groups comprised neonate optimal males (*n* = 23), neonate low males (*n* = 19), neonate low-optimal males (*n* = 6), neonate optimal-low males (*n* = 12), neonate optimal females (*n* = 22), neonate low females (*n* = 18), neonate low-optimal females (*n* = 9), neonate optimal-low females (*n* = 9), juvenile optimal males (*n* = 13), juvenile low males (*n* = 10), juvenile low-optimal males (*n* = 9), juvenile optimal-low males (*n* = 10), juvenile optimal females (*n* = 11), juvenile low females (*n* = 10), juvenile low-optimal females (*n* = 8), juvenile optimal-low females (*n* = 11), adolescent optimal males (*n* = 8), adolescent low males (*n* = 7), adolescent low-optimal males (*n* = 8), adolescent optimal-low males (*n* = 8), adolescent optimal females (*n* = 9), adolescent low females (*n* = 7), adolescent low-optimal females (*n* = 8), adolescent optimal-low females (*n* = 9). To prevent any bias from specific litter effects, no more than two pups per sex per litter were assigned to the same age group.

Following delivery, all mothers and pups were provided the optimal (900 mg vitamin C/kg feed) diet to ensure that any observed effects in the offspring were exclusively attributed to prenatal vitamin C depletion. Mothers and pups were initially housed together in a cage for the first week and were later group-housed in a nursery pen with other mothers and pups until weaning on day 21. Subsequently, offspring were housed in single-sex pens. Offspring were weighed daily for the first week and then weekly until euthanasia.

### 2.4. Oral Glucose Tolerance Tests

Oral glucose tolerance tests (OGTTs) were performed on juvenile offspring at weaning (day 21) using our previously published protocol [3,38]. Animals fasted for at least 4 h prior to a baseline glucose reading and blood collection. Dextrose (1000 mg/kg, Dextrose 50%, Biomed Ltd., Auckland, New Zealand) was administered orally with serial glucose readings and blood samples collected at 30, 60, 120, and 180 min post-glucose dose.

### 2.5. Cortisol Enzyme-Linked Immunosorbent Assay (ELISA) 

Saliva samples, obtained by allowing animals to chew on the tip of a cotton bud, were collected from both dams and pups on the day of delivery/birth (day zero). Subsequently, samples were collected from offspring on days 7 and 25. The Salimetrics Salivary Cortisol Assay (Salimetrics Inc., State College, PA, USA) was employed to measure cortisol concentrations in guinea pig saliva samples, following the manufacturer’s instructions. The assay demonstrated a sensitivity range of 0.012–3.0 μg/dL, with inter- and intra-assay coefficients of variance recorded at 8.6% and 5.1%, respectively.

### 2.6. Euthanasia and Tissue Collection

Neonates fasted for at least 2 h and juveniles and adolescents fasted for at least 4 h prior to anaesthetisation by Isoflurane inhalation (Attane^TM^, Bayer Australia Ltd., Pymble, NSW, Australia). Upon the disappearance of voluntary reflexes, an intracardial blood sample was collected and euthanasia was achieved by exsanguination and subsequent decapitation. Organs were dissected, weighed, and processed for downstream analyses.

### 2.7. Statistical Analysis 

The analysis of all data was conducted using GraphPad Prism software (version 9.2.0). All tests were two-tailed, with significance considered at *p* < 0.05. Means and standard deviations (SD) were employed for continuous variables analysis, while counts with proportions were used for categorical variables analysis. Normality was assessed using the D’Agostino Pearson test.

For maternal data, group differences were evaluated using one-way ANOVA or Fisher’s exact test for proportions. For offspring data, group differences within sexes were assessed using one-way ANOVA. Post hoc tests with Tukey’s correction for multiple comparisons were carried out when the ANOVA yielded *p* < 0.05. For data that did not exhibit normal distribution, the Kruskal–Wallis test with Dunn’s correction for multiple comparisons was employed. The *p*-values for multiple comparisons are reported in the text, while the overall model *p*-values are provided in the Supplementary Materials. Weight gain trajectories were analysed using repeated measures two-way ANOVA.

## 3. Results

### 3.1. Maternal Physiology 

Herein, we compare dams from the low-optimal and optimal-low groups with each other and with dams from the optimal and low groups, using data from our previously published study [36].

#### 3.1.1. Maternal Characteristics

Mean weights at study enrolment and mean weights and ages at mating are presented in Table 1 (see also Supplementary Table S1). No significant differences were observed between groups.

#### 3.1.2. Pregnancy Weight Gain

To observe any differences in weight gain during pregnancy, dams were weighed weekly. We have previously reported that dams with consistently optimal vitamin C intakes show greater weight gain during pregnancy compared to dams with consistently low vitamin C intakes [36]. However, the difference in pregnancy-associated weight gain between the optimal and low dams was due to a positive correlation between litter size (*n* pups) and weight gain during pregnancy; when comparisons were made within dams carrying the same number of pups, no significant differences were observed [36].

**Figure 1:**
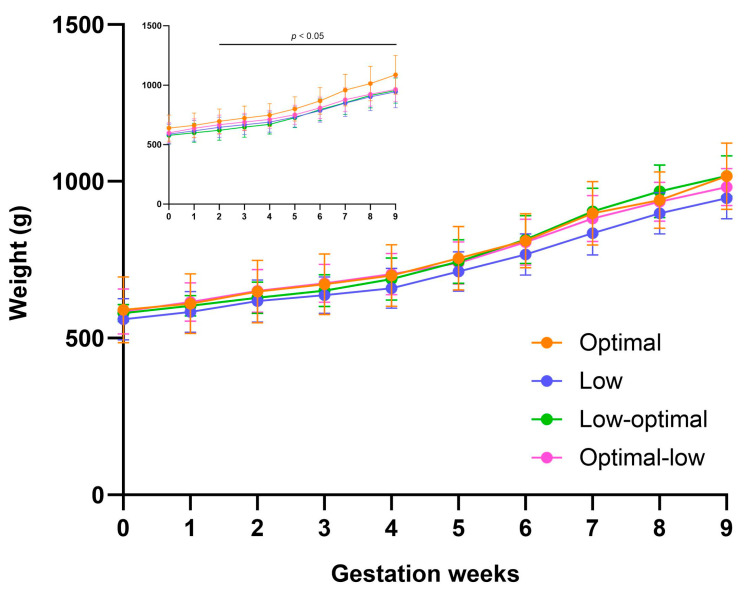
Pregnancy weight gain. The graph represents average weight gain during pregnancy (mating to week of delivery) ± SD. Insert figure includes total dams and main figure includes dams carrying three pups. Optimal dams (orange, *n* = 30 insert, *n* = 11 main), low dams (purple, *n* = 32 insert, *n* = 10 main), low-optimal dams (green, *n* = 22 insert, *n* = 6 main), optimal-low dams (pink, *n* = 21 insert, *n* = 9 main). Data were analysed using repeated measures mixed-effects two-way ANOVA.

Similarly, when compared to dams who had low vitamin C intake during the first trimester (low-optimal group), the optimal dams showed greater weight gain. This was apparent from the second week of pregnancy (week two; *p* = 0.0323, week three; *p* = 0.0242, week four; *p* = 0.0146, week five; *p* = 0.0303, week six; *p* = 0.0553, week seven; *p* = 0.0091, week eight; *p* = 0.0310, week nine; *p* = 0.0046) (Figure 1 Insert and Supplementary Table S2). When compared to dams who had low vitamin C intake during mid–late pregnancy (optimal-low group), the optimal dams had significantly greater weights during the final week of pregnancy (week nine; *p* = 0.0096). However, when comparisons were made within dams carrying the same number of pups (*n* = 3 pups) (Figure 1 Main), no significant differences were observed.

#### 3.1.3. Pregnancy Outcomes

To evaluate the impact of vitamin C intake on overall pregnancy and perinatal outcomes, various parameters were examined, including rates of pregnancy loss/miscarriage, foetal reabsorption, premature delivery, and stillbirth. Additionally, assessments were made on litter size, gestational age (GA) of pups at delivery, litter birth weight, and pup sex ratios (Table 2 and Supplementary Table S3).

Delivery of pups prior to GA 62 (considering term delivery occurs around GA 69) typically results in stillbirth or early neonatal mortality, requiring neonatal resuscitation and extensive intervention for survival. In this study, spontaneous delivery before GA 62 was categorized as a miscarriage. Litters born between GA 62 and GA 66, although potentially viable without intervention, were categorized as premature.

The identification of foetal reabsorption was based on a higher number of pups detected by ultrasound compared to the actual number delivered. This phenomenon was accompanied by significant maternal weight loss or weight stagnation during pregnancy and/or the delivery of partially reabsorbed pups.

Due to the risk of cannibalism of underdeveloped guinea pig pups, instances of miscarriages and premature deliveries were excluded from the analysis of litter size (number of pups). Because litter size impacts both pregnancy duration and pup birth weights, the GA of pups at delivery and average litter birth weight were analysed only for term litters of 2–4 pups, which is typical for guinea pigs †.

The optimal-low dams had the lowest stillbirth rate (# of pups) at 1.6%. This was statistically significant when compared to the low (13.1%, *p* = 0.0141) dams but not the optimal (5.9%, *p* = 0.2580) or low-optimal (3.9%, *p* = 0.5905) dams. The low-optimal dams delivered significantly fewer offspring in terms of litter size (*n* pups) when compared to the optimal (*p* = 0.0002) dams but not the low (*p* = 0.3747) or optimal-low (*p* = 0.0840) dams. The average birth weight of the low-optimal litters was increased compared to the optimal (*p* = 0.0151) and optimal-low (*p* < 0.0001) litters but not the low (*p* = 0.0646) litters. The average birth weight of the optimal-low litters was additionally decreased compared to the low (*p* = 0.0224) litters but not the optimal (*p* = 0.0917) litters. The cumulative adverse pregnancy outcome rate which included miscarriages, foetal reabsorption, premature deliveries, and stillbirth (# of pregnancies) was lower in the optimal-low group (27.3%) when compared to the low (61.1%, *p* = 0.0160) group but not the optimal (32.4%, *p* = 0.7718) or low-optimal (31.8%, *p* > 0.9999) group. Other pregnancy outcomes were unaffected by maternal vitamin C status.

#### 3.1.4. Maternal Salivary Cortisol Concentrations

**Figure 2:**
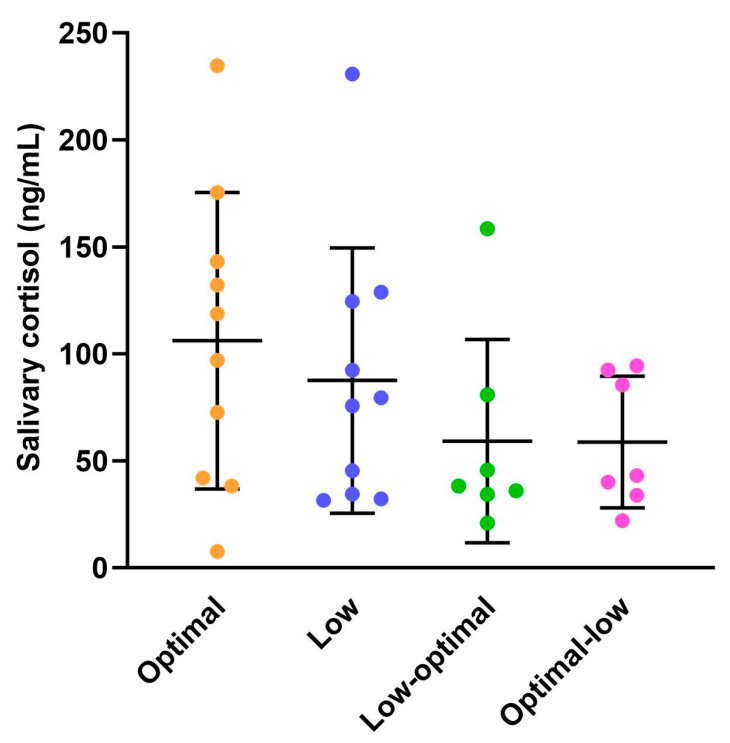
Postpartum salivary cortisol concentrations in dams. Optimal (orange, *n* = 10), low (purple, *n* = 10), low-optimal (green, *n* = 7), and optimal-low (pink, *n* = 7). Data are presented as group means ± SD and were analysed using one-way ANOVA.

Salivary cortisol concentrations in dams were evaluated on the day of delivery, as depicted in Figure 2 (see also Supplementary Table S4). There was no significant impact of vitamin C status during pregnancy on postpartum salivary cortisol concentrations. This suggests that there were no apparent birth complications leading to heightened stress during labour or delivery for dams in any of the groups.

### 3.2. Offspring Physiology 

Herein, we compare neonates from the low-optimal and optimal-low groups with each other and with neonates from the optimal and low groups, using data from our previously published study [36].

#### 3.2.1. Neonate Characteristics

On the day of delivery/birth (day zero), body weights and measurements were recorded and are displayed below in Table 3 (see also Supplementary Table S5). The birth weight was significantly affected by maternal vitamin C status for the offspring of both sexes. For females, pups born to low-optimal mothers had significantly greater birth weights when compared to females from optimal (*p* = 0.0014), low (*p* = 0.0165), and optimal-low (*p* < 0.0001) mothers. The low-optimal males also had greater birth weights, and this was statistically significant when compared to males from optimal-low (*p* = 0.0034) mothers but not optimal (*p* = 0.0903) or low (*p* = 0.4339) mothers. Birth length (CRL) was similarly affected; low-optimal females were significantly longer than their optimal (*p* = 0.0011), low (*p* < 0.0001), and optimal-low (*p* < 0.0001) counterparts. Low-optimal males were significantly longer than the optimal-low (*p* = 0.0068) males but not the optimal or low (*p* = 0.0797 for both) males. Optimal-low females had shorter hind limbs (HL) at birth compared to their optimal (*p* = 0.0066) and low-optimal (*p* = 0.0232) but not low (*p* = 0.3414) counterparts. The ponderal index (PI), an estimate of adiposity in relation to length, was calculated using the following formula: weight (kg)/length (m)^3^. The sum of a guinea pig’s CRL and HL was used as an approximation of the animal’s height. Interestingly, the average PI-derived adiposity at birth was decreased in pups from low-optimal mothers despite their increased birth weights and lengths. This was statistically significant when compared to pups from the low (males; *p* = 0.0485 and females; *p* = 0.0033) group but not the optimal (males; *p* > 0.9999 and females; *p* > 0.9999) or optimal-low (males; *p* > 0.9999 and females; *p* = 0.4959) group. No other significant differences were identified.

At the time of neonate tissue collection (within 24 h of delivery), organ weights were recorded and are displayed below in Table 4. Maternal vitamin C status had a significant effect on the relative adrenal, testes, kidney, and liver weights and the brain-to-liver ratio (BLR) for male offspring. Males from low-optimal mothers had decreased adrenal weights when compared to males from optimal (*p* = 0.0076) mothers but not low (*p* = 0.1179) or optimal-low (*p* > 0.9999) mothers. Similarly, optimal-low males had decreased adrenal weights when compared to optimal (*p* = 0.0107) males but not low (*p* = 0.1968) males. The same trend was observed for testis weights; low-optimal males had decreased testis weights when compared to optimal (*p* = 0.0107) males but not low (*p* = 0.1843) or optimal-low (*p* < 0.9999) males. Similarly, optimal-low males had decreased testis weights when compared to their optimal (*p* = 0.0227) counterparts but not their low (*p* = 0.5006) counterparts. Kidney weights were significantly decreased in males born to optimal-low mothers when compared to males from low-optimal (*p* = 0.0344) mothers but not optimal (*p* = 0.6433) or low (*p* = 0.9719) mothers. Optimal-low males also had decreased liver weights when compared to their optimal (*p* = 0.0191) and low-optimal (*p* = 0.0102) counterparts but not their low (*p* = 0.1284) counterparts. The brain-to-liver ratio (BLR) in optimal-low males was correspondingly increased and this was statistically significant when compared to males from optimal (*p* = 0.0377), low (*p* = 0.0164), and low-optimal (*p* = 0.0201) mothers. Maternal vitamin C status had no significant effect on organ weights at birth for female offspring.

**Figure 3:**
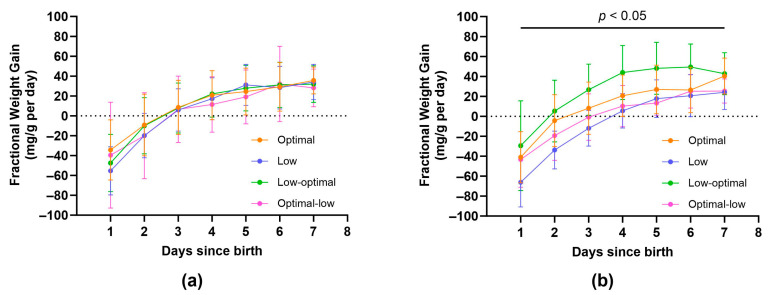
Fractional weight gain in male (**a**) and female (**b**) offspring during the neonatal period. Optimal offspring (orange, *n* = 21 males, *n* = 20 females), low offspring (purple, *n* = 17 males, *n* = 17 females), low-optimal offspring (green, *n* = 17 males, *n* = 16 females), optimal-low offspring (pink, *n* = 18 males, *n* = 20 females). Data are presented as group means ± SD and were analysed using repeated measures mixed-effects two-way ANOVA.

All pups allocated to the juvenile or adolescent groups were closely monitored for the first week of life. An important indicator of health and wellbeing during the neonatal period is daily fractional weight gain (Figure 3a,b and Supplementary Table S6). Maternal vitamin C status had a significant effect on fractional weight gain for female offspring (Figure 3b). Females born to low-optimal mothers exhibited increased fractional weight gain when compared to females from low and optimal-low mothers during the first seven days of life (postnatal day one; low-optimal vs. low, *p* = 0.0423, day two; low-optimal vs. low, *p* = 0.0012, day three; low-optimal vs. low, *p* = 0.0005 and low-optimal vs. optimal-low, *p* = 0.0205, day four; low-optimal vs. low, *p* = 0.0113 and low-optimal vs. optimal-low, *p* = 0.0278, day five; low-optimal vs. low, *p* = 0.0449 and low-optimal vs. optimal-low, *p* = 0.0159, day six; low-optimal vs. low, *p* = 0.0386, day seven; low-optimal vs. low, *p* = 0.0486 and low-optimal vs. optimal-low, *p* = 0.0425). Maternal vitamin C status had no significant effect on daily fractional weight gain for male offspring.

#### 3.2.2. Weanling Characteristics 

On the day of weaning (day 21), body measurements were recorded and are displayed below in Table 5 (see also Supplementary Table S7). Maternal vitamin C status had no significant effect on the linear growth trajectory (CRL), HL growth trajectory, or hock-toe (HT) growth trajectory from birth to weaning for offspring of either sex.

#### 3.2.3. Juvenile and Adolescent Characteristics 

At the time of juvenile tissue collection (day 28), organ weights were recorded and are displayed below in Table 6 (see also Supplementary Table S8). Maternal vitamin C status had a significant effect on the relative adrenal weights for male offspring. Low-optimal males exhibited increased adrenal gland weights, and this was statistically significant when compared to the optimal (*p* = 0.0345) and low (*p* = 0.0358) males but not the optimal-low (*p* = 0.1500) males. Maternal vitamin C status had no significant effect on organ weights at 28 days of age for female offspring.

**Figure 4:**
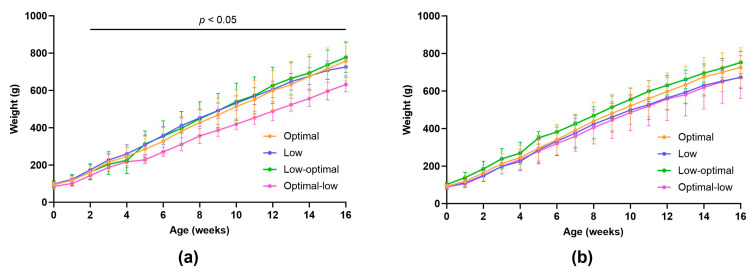
Weight growth rate in male (**a**) and female (**b**) offspring from birth to four months. Data are pooled from pups randomised at birth to the juvenile (up to week four) and adolescent groups. Optimal offspring (orange, *n* = 21 males, *n* = 20 females), low offspring (purple, *n* = 17 males, *n* = 17 females), low-optimal offspring (green, *n* = 17 males, *n* = 16 females), optimal-low offspring (pink, *n* = 18 males, *n* = 20 females). Data are presented as group means ± SD and were analysed using repeated measures mixed-effects two-way ANOVA.

To observe any differences in offspring weight gain, all pups allocated to the juvenile or adolescent groups were weighed weekly until euthanasia on day 28 (juveniles) or at 4 months (adolescents) (Figure 4a,b and Supplementary Table S9). Maternal vitamin C status had a significant effect on the weight gain trajectory in male offspring (Figure 4a). Optimal-low males showed significantly reduced weight gain from the second week of life through adolescence (week two; optimal-low vs. low, *p* = 0.0112, week three; optimal-low vs. low, *p* = 0.0177, week four; optimal-low vs. low, *p* = 0.0257, week five; optimal-low vs. low, *p* = 0.0213 and optimal-low vs. low-optimal, *p* = 0.0455, week six; optimal-low vs. low, *p* = 0.0079, week seven; optimal-low vs. low, *p* = 0.0016, week eight; optimal-low vs. low, *p* = 0.0053, week nine; optimal-low vs. low, *p* = 0.0010, week 10; optimal-low vs. low, *p* = 0.0003 and optimal-low vs. low-optimal, *p* = 0.0448, week 11; optimal-low vs. low, *p* = 0.0010 and optimal-low vs. low-optimal, *p* = 0.0446, week 12; optimal-low vs. low, *p* = 0.0034 and optimal-low vs. low-optimal, *p* = 0.0232, week 13; optimal-low vs. low, *p* = 0.0005 and optimal-low vs. low-optimal, *p* = 0.0103, week 14; optimal-low vs. low, *p* = 0.0012 and optimal-low vs. low-optimal, *p* = 0.0114, week 15; optimal-low vs. low, *p* = 0.0051 and optimal-low vs. low-optimal, *p* = 0.0054, week 16; optimal-low vs. low, *p* = 0.0087 and optimal-low vs. low-optimal, *p* = 0.0043. Maternal vitamin C status had no significant effect on the weight gain trajectory for female offspring.

At the time of adolescent tissue collection (four months), organ weights were recorded and are displayed below in Table 7 (see also Supplementary Table S10). Maternal vitamin C status had a significant effect on the relative brain, subcutaneous fat, and visceral fat weights for male offspring. The optimal-low males exhibited increased brain weights when compared to the optimal (*p* = 0.0119) males but not the low (*p* = 0.0612) or low-optimal (*p* = 0.0869) males. The optimal-low males also had decreased subcutaneous fat weights compared to the low (*p* = 0.0355) males but not the optimal (*p* = 0.3248) or low-optimal (*p* = 0.0780) males and decreased visceral fat weights compared to the low-optimal (*p* = 0.0306) males but not the optimal (*p* = 0.8203) or low (*p* = 0.3349) males. Maternal vitamin C status had no significant effect on organ weights at four months of age for female offspring.

#### 3.2.4. Oral Glucose Tolerance Tests 

**Figure 5:**
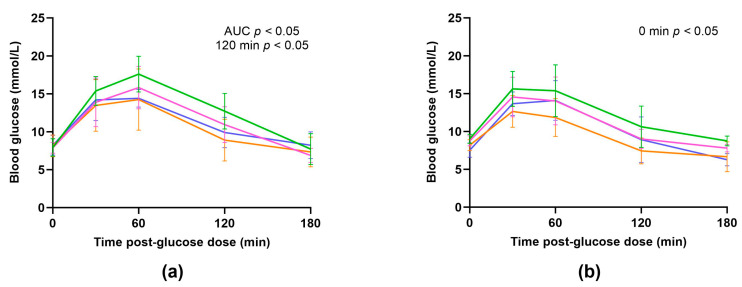
Glucose response in male (**a**) and female (**b**) offspring at weaning on day 21. Optimal offspring (orange, *n* = 8 males, *n* = 8 females), low offspring (purple, *n* = 6 males, *n* = 8 females), low-optimal offspring (green, *n* = 6 males, *n* = 5 females), and optimal-low offspring (pink, *n* = 5 males, *n* = 9 females). Data are presented as group means ± SD and were analysed within sex using one-way ANOVA.

Oral glucose tolerance tests (OGTT’s) were performed on offspring from all four dietary groups at weaning (day 21) to examine any potential effect of maternal vitamin C intake on the offspring’s glucose response. Serial blood glucose readings were plotted against time (Figure 5a,b and Supplementary Table S11) and the absolute peak, delta-peak (difference between baseline blood glucose and peak blood glucose concentrations), time to peak, and the area under the curve (AUC) were analysed for each individual animal (Table 8). The AUC was generated to quantify the total increase in blood glucose concentration during the OGTT (mmol/L × minutes).

There was no significant effect of maternal vitamin C status on the absolute peak, delta-peak, or time to peak for the offspring of either sex. However, there was a significant effect of maternal diet on the AUCs generated from the glucose response curves in males. The AUC (total increase in blood glucose concentration during the OGTT) was significantly greater in males born to low-optimal mothers when compared to males from optimal (*p* = 0.0152) and low (*p* = 0.0373) mothers but not optimal-low (*p* = 0.2189) mothers.

At specific time points during the OGTT, a few significant differences were observed in glucose concentrations between groups. At baseline (0 min), females born to mothers who swapped diets during their pregnancies (low-optimal and optimal-low groups) had increased blood glucose concentrations when compared to females from low (*p* = 0.0178 and *p* = 0.0130, respectively) mothers but not optimal (*p* = 0.2936 and *p* = 0.4103, respectively) mothers. At 120 min post-glucose dose, males from low-optimal mothers had significantly increased blood glucose concentrations compared to males from optimal (*p* = 0.0379) mothers but not low (*p* = 0.2141) or optimal-low (*p* = 0.6262) mothers. No other significant differences were identified.

Of the 55 animals that underwent glucose tolerance testing, three did not return to their baseline level within the timeframe of the OGGT (180 min). These animals were one low male, one low-optimal male, and one low female. These animals were included in all OGGT analyses as their exclusion did not affect the statistical results.

#### 3.2.5. Offspring Salivary Cortisol Concentrations

**Figure 6:**
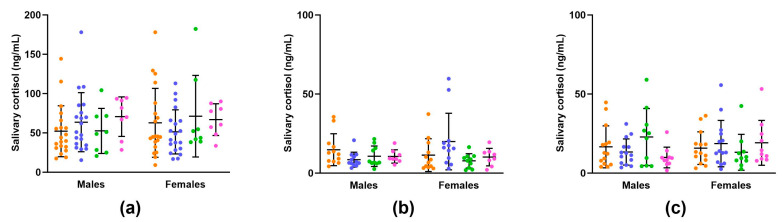
Salivary cortisol concentrations in offspring on day zero (**a**), seven (**b**), and twenty-five (**c**). On day zero, optimal offspring (orange, *n* = 20 per sex), low offspring (purple, *n* = 20 per sex), low-optimal offspring (green, *n* = 8 per sex), and optimal-low offspring (pink, *n* = 8 per sex). On day seven, optimal offspring (orange, *n* = 12 per sex), low offspring (purple, *n* = 12 per sex), low-optimal offspring (green, *n* = 10 per sex), and optimal-low offspring (pink, *n* = 8 per sex). On day 25, optimal offspring (orange, *n* = 14 per sex), low offspring (purple, *n* = 14 per sex), low-optimal offspring (green, *n* = 10 per sex), and optimal-low offspring (pink, *n* = 10 per sex). Data are presented as group means ± SD and were analysed within sex using one-way ANOVA.

Salivary cortisol was measured in pups on days zero, seven, and twenty-five (Figure 6a–c). There was no significant effect of maternal vitamin C status on salivary cortisol concentrations for offspring of either sex at any of the timepoints assessed. A consistent finding across all dietary groups and both sexes was that baseline cortisol concentrations at birth (day zero) were significantly increased compared to baseline cortisol concentrations at day seven and day twenty-five (Supplementary Table S12). This is to be expected given that cortisol is required for lung maturation and surfactant production occurring shortly before birth [23].

#### 3.2.6. Offspring Mortality

Two animals died spontaneously, and one animal was euthanised during the study period, as shown in Table 9.

## 4. Discussion

This study provides compelling evidence of the timing-specific impact of maternal vitamin C intake on pregnancy outcomes and the timing- and sex-specific impacts on offspring physiology and metabolic function in guinea pigs. Guinea pigs serve as an excellent and clinically relevant model for studying human pregnancy and foetal development, as previously reviewed [39]. Moreover, they represent a natural model of vitamin C dependency [9].

We found that pregnancies characterised by low vitamin C intake specifically during mid–late pregnancy (optimal-low group) exhibited the lowest stillbirth rate, accounting for only 1.6% of the total pups born. These pregnancies also resulted in the smallest average litter birth weight. This lower birth weight implies smaller foetal size, which, in turn, can reduce the risk of labour complications and subsequent stillbirth [40]. Among pregnancies characterised by low vitamin C intake specifically during the first trimester (low-optimal group), we found that the dams gave birth to significantly fewer offspring (*n* pups born per litter). This finding is consistent with our previous data, which revealed that low vitamin C intake during preconception and pregnancy significantly reduces fecundity [36].

A key finding from our neonate biometric data is that male pups born to mothers who had low vitamin C intake specifically during mid–late pregnancy (optimal-low) displayed characteristic features of intrauterine growth restriction (IUGR). Clinically, IUGR is classified into symmetrical (type I) and asymmetrical (type II) forms [41,42]. Symmetrical IUGR constitutes 20–30% of all cases and is typically attributed to TORCH infections (toxoplasmosis, rubella, cytomegalovirus, and others) during the first trimester or chromosomal abnormalities, such as aneuploidy [41,43,44]. Asymmetrical IUGR, which accounts for 70–80% of all cases, usually results from a failure of the placenta to supply adequate nutrients and oxygen to the rapidly growing late-gestation foetus. This can occur due to maternal malnutrition or maternal hypertension in the second or third trimesters [41,45,46]. Foetuses with symmetrical IUGR are proportionally small, while foetuses with asymmetrical IUGR are disproportionate, with sparing of normal brain size at the expense of other organs, such as the liver, resulting in an increased brain-to-liver ratio (BLR) [42].

We observed a significant increase in the BLR among male pups from optimal-low mothers compared to male pups in the optimal, low, and low-optimal groups, indicating asymmetrical IUGR. Additionally, these males had reduced liver, kidney, adrenal, and testes weights. This is in line with clinical observations that an adverse in utero environment, characterised by limited nutrient availability during mid–late gestation, can result in asymmetrical IUGR [41,42,45]. Furthermore, optimal-low females exhibited decreased hind limb lengths at birth, indicating some mild growth restriction of the limbs. Interestingly, the same features of IUGR were not apparent in pups from mothers with consistently low vitamin C intakes during pregnancy. These findings suggest that an abrupt change in the foetal nutritional environment, specifically the removal of vitamin C, may be more deleterious to foetal growth than a consistent nutritional environment, even if vitamin C availability is low.

Another key finding from the neonate biometric data is that female pups born to mothers who had low vitamin C intake specifically during the first trimester (low-optimal) had higher birth weights and increased birth lengths compared to females from the optimal, low, and optimal-low groups. The low-optimal males also had increased birth weights and lengths, but this was only statistically significant when compared to the optimal-low males, who displayed characteristic features of foetal growth restriction and, therefore, there may not be an effect of being constitutionally bigger, per se.

This observation of increased size at birth, primarily in females, could be partly due to the fact that low-optimal dams gave birth to smaller litters (*n* pups), resulting in less growth restriction for the individual pups in utero and ultimately leading to larger sizes at birth. However, the female pups from low-optimal mothers were also significantly larger than the female pups from mothers with consistently low vitamin C intakes who produced similarly small litters (*n* pups). This suggests that there is an additional mechanism at play that is influencing the size difference.

Similar to what occurs postnatally in low-birth-weight human infants [47,48,49], this finding could reflect a period of accelerated catch-up growth occurring in the second half of gestation, as if to mitigate the adverse effects of low vitamin C availability during the first trimester. Furthermore, the larger at birth low-optimal females also exhibited accelerated growth during the neonatal period (Figure 3). To conclusively determine if compensatory growth, beginning in gestation and continuing in early postnatal life, was indeed a factor in our cohort, a foetal ontogeny study is required to compare the growth rates of ‘normal’ foetuses to the growth rates of vitamin C-depleted foetuses.

Much like human foetuses, guinea pig foetuses accumulate brown and white adipose tissue during late gestation, with a total fat content of ~14% at birth [50], similar to the human infant (~10%) [51]. Interestingly, average adiposity (as assessed by the ponderal index) was decreased in the low-optimal pups despite their increased birth weights and lengths. However, by weaning, there were no significant differences in average adiposity between groups for either sex, indicating a period of catch-up fat accumulation occurring during early postnatal life, a phenotype that is widely reported to be associated with an increased risk of adult cardiometabolic disease [49,52,53,54,55].

Overall, our neonate biometric data indicate that maternal vitamin C status has significant timing- and sex-specific impacts on birth weight, birth length, organ weights at birth, and subsequent neonatal growth. Specifically, males exposed to vitamin C depletion during mid–late gestation exhibited foetal growth restriction. Conversely, females exposed to vitamin C depletion during early gestation were born larger and exhibited accelerated growth during the neonatal period. These observations align with a long-observed phenomenon wherein female newborns generally have better outcomes than their male counterparts. A prominent theory underpinning this phenomenon is the existence of divergent growth and development strategies in utero, which place males at an increased risk if conditions are sub-optimal [56,57]. Generally, males grow faster and larger in utero and consequently have a higher demand for nutrients and oxygen supplied through the placenta [58,59]. As a result, they are at an increased risk of undernutrition and may be more vulnerable to specific nutritional deficiencies [56,57,58,59]. Our findings suggest that male foetuses are particularly vulnerable to the adverse effects of vitamin C depletion during mid–late gestation, while female foetuses appear to possess compensatory mechanisms enabling them to better cope with or recover from vitamin C depletion, especially if it occurs in early gestation and is temporary, with improved nutrient supply starting from the end of the first trimester.

Considering the longitudinal outcomes for these offspring, the IUGR optimal-low males went on to exhibit consistently reduced weight gain throughout their lifespan (Figure 4). Notably, at four months of age, they displayed significantly increased relative brain weights and significantly decreased subcutaneous and visceral fat weights. This persistent pattern of brain sparing at the expense of overall body growth may indicate a programmed dysregulation of the somatotropic axis. The somatotropic axis, consisting of growth hormone (GH), insulin-like growth factors (IGF-I and -II), and their associated proteins and receptors, is functional in utero and plays a critical role in regulating postnatal nutritional status, energy balance, and growth [60,61,62]. Alterations to the somatotropic axis and changes in the circulating concentrations of its components have been widely reported in offspring born to poorly nourished mothers across various species [62,63,64,65,66,67,68]. Our findings suggest that the developmental programming and long-term function of the somatotropic axis may be particularly sensitive to nutritional perturbations that occur in mid–late gestation during the most rapid phase of overall foetal growth.

For the low-optimal offspring, a key finding at juvenility (28 days of age) was that males had significantly increased relative adrenal weights. Adrenal enlargement is a cardinal feature of congenital adrenal hyperplasia (CAH). Congenital adrenal hyperplasia is a genetic disorder in which specific enzymes, most commonly 21-hydroxylase, which are required for producing adrenal hormones such as cortisol do not function properly, resulting in cortisol deficiency [69]. On the other hand, adrenal enlargement can also occur in conditions associated with excessive cortisol production and hyperactivity of the hypothalamic–pituitary–adrenal (HPA) axis, such as Cushing’s syndrome [70]. In our study, we measured baseline concentrations of salivary cortisol at three specific timepoints: twice during the neonatal period at day zero and day seven, and once during the human equivalent of mid-childhood at day twenty-five. The collection of saliva is non-invasive, and studies consistently report high correlations between salivary cortisol concentrations and those found in serum and various tissues [71,72]. Despite the observed adrenal enlargement in the low-optimal males, there were no discernible differences in basal cortisol concentrations at any of the three timepoints assessed (Figure 6). This suggests that the adrenal enlargement may be due to factors other than changes in cortisol production or regulation.

Due to the involvement of the HPA axis in the regulation of metabolism, it is unsurprising that metabolic dysfunction is a common occurrence in disorders of the adrenal glands. For instance, individuals with CAH have a higher prevalence of obesity, hypertension, and diabetes [73,74]. Similarly, those with Cushing’s syndrome have an increased incidence of hyperglycaemia, insulin resistance, and excess visceral adiposity [75]. To investigate the potential impact of maternal vitamin C intake on offspring metabolic function, namely glucose homeostasis and diabetes risk, we conducted oral glucose tolerance tests (OGTTs) on pups at weaning. Guinea pigs are an appropriate species for studying metabolic function due to their similarities to humans in terms of glucose metabolism, where glucose uptake occurs in a dose-dependent, insulin-regulated manner [39,76]. Moreover, in a diet-induced model of type 2 diabetes in guinea pigs, animals developed impaired glucose tolerance and hyperinsulinemia, mirroring what occurs in prediabetic humans [77]. There is also evidence of foetal programming of glucose metabolism by maternal nutrition in guinea pigs [78], as observed in humans [7].

During the OGTT conducted at weaning, we found that female offspring born to mothers who had low vitamin C intake specifically during the first trimester (low-optimal) had increased fasting blood glucose concentrations. Meanwhile, their male counterparts exhibited a greater total increase in blood glucose concentration during the OGTT and had significantly elevated blood glucose concentrations 120 min post-glucose dose, suggesting impaired glucose tolerance and an increased susceptibility to diabetes in adulthood for both sexes. We have not performed a reciprocal analysis of plasma insulin, which may offer more insight into the potential differences in glucose homeostasis and metabolic dysfunction.

An important connection to note is that offspring displaying metabolic phenotype indicators also demonstrated accelerated perinatal weight gain and fat accumulation. Clinically, growth rate stands as one of the best indices of an infant’s immediate physical health and has long been a top priority in paediatric practice. However, the long-term cost of faster growth in infancy manifests as an increased risk of noncommunicable disease in later life. This ‘grow now, pay later’ concept finds support in a wealth of evidence in humans and has been experimentally reproduced using animal models [55,79,80]. For instance, in humans, a rapid increase in BMI during the first year has been linked to lower insulin sensitivity, higher cholesterol, obesity, and increased visceral fat—all key factors that contribute to an elevated metabolic risk profile [52,54,55,81,82,83,84].

Our OGTT findings provide a fresh perspective on unravelling the coupling mechanisms between nutritional inadequacies in early life and later outcomes. Specifically, we suggest timing-specific mechanisms where foetal exposure to low vitamin C availability, specifically during early gestation, programs a dysregulation of glucose metabolism that persists until at least an early childhood-equivalent age. This may resemble the mismatch theory of foetal programming, which proposes that a developing foetus adapts in anticipation of its future postnatal environment based on prenatal environmental cues, such as maternal nutrient availability [85,86]. When there is a mismatch between the anticipated and actual future environments, previously beneficial adaptations can become maladaptive [85].

In vivo, vitamin C exists in two forms: ascorbate (ASC), the reduced form, and dehydroascorbic acid (DHA), the oxidised form, with the former being the most predominant [35,87]. Most cellular uptake occurs as ASC via sodium-dependent vitamin C transporters (SVCTs). However, in some cells of the body, such as erythrocytes, SVCTs are absent. In these cells, DHA must compete with glucose for facilitated diffusion via the glucose transporters (GLUTs) before being converted to ASC intracellularly [35,87]. Considering low maternal vitamin C intake, it is possible that insufficient vitamin C availability during early gestation may trigger an adaptive protection mechanism or salvage pathway. For example, increased insulin sensitivity, which leads to reduced blood glucose levels, could promote the uptake of DHA via the GLUTs, thereby increasing the intracellular pool of ASC. Over time, this could lead to insulin resistance, increasing the risk of hyperglycaemia, impaired glucose tolerance, and type 2 diabetes [88,89].

Overall, our research has identified two distinct and persistent phenotypic presentations of reduced vitamin C availability at different developmental stages during pregnancy. Low levels during early development were associated with accelerated perinatal weight gain and fat accumulation and a metabolic syndrome-related phenotype at a childhood-equivalent age. In contrast, low levels during late development were associated with IUGR and long-term growth abnormalities, including reduced weight gain and fat deposition throughout childhood and adolescence.

The developmental programming of these phenotypes may, in part, be mediated by epigenetic modifications of transcription factors and regulatory genes that influence metabolism, energy balance, and growth rate. In humans, a study of the 1944–45 Dutch Hunger Winter revealed that prenatal exposure to famine, specifically during early gestation, resulted in less DNA methylation of the IGF-II gene compared to sex-matched siblings who were either unexposed or exposed during late gestation [90]. In mice, IUGR pups demonstrate sex-specific changes in placental DNA methylation, with the effects being more pronounced in male offspring [91].

The ten-eleven translocation (TET) enzymes (TET1, TET2, TET3) provide a pertinent example of how variations in maternal nutrient availability might affect the process of development through persistent epigenetic changes. These enzymes are pivotal regulators of the epigenome during early mammalian development, and their catalytic activity is largely dependent on the bioavailability of vitamin C [34,92,93]. In a study by Kawahori et al. that employed a Gulo-/- knockout mouse model of vitamin C dependency, the authors showed that offspring born to vitamin C-deficient mothers exhibited DNA hypermethylation in their livers. Specifically, they identified hypermethylation in gene pathways involved in fatty acid metabolism and glycerolipid metabolism, suggesting a possible link between inadequate maternal vitamin C, epigenetic alterations, and the development of metabolic abnormalities in the offspring. We postulate that similar mechanisms may be in effect in our study, with low maternal vitamin C intake influencing the expression of genes involved in the complex interaction of genetic, hormonal, and environmental factors that regulate growth and metabolism across the lifespan. While the current investigation focuses on the broader physiological outcomes of prenatal vitamin C depletion, it is imperative that future work is conducted to identify the molecular mechanistic underpinnings of our findings. This will involve comprehensive biochemical analyses, including gene, protein, and DNA methylation sequencing.

## 5. Conclusions

In conclusion, we have presented compelling evidence regarding the adverse effects of low maternal vitamin C intake during pregnancy on offspring’s long-term outcomes—in a species that shares the same dietary requirements for vitamin C as humans. We found that the timing of vitamin C depletion during pregnancy can result in divergent effects on offspring. Specifically, low vitamin C intake during early pregnancy accelerated foetal and neonatal growth in female offspring and altered glucose homeostasis in the offspring of both sexes at an age equivalent to early childhood. In contrast, when maternal vitamin C intake was low during mid-late pregnancy, it resulted in foetal growth restriction and reduced weight gain throughout the lifespan, primarily affecting male offspring. These sex-specific effects on offspring growth not only highlight the complex interplay between maternal nutrition and offspring outcomes but may be important to help guide future sex-specific therapeutic strategies or preventive measures for pregnant women, their unborn babies, and newborns. Future studies should investigate outcomes for offspring who also experience postnatal vitamin C depletion, which could exacerbate the adverse effects we have reported. This aspect is particularly relevant for exclusively breastfed infants born to mothers with low vitamin C status or for young children in regions where access to diverse vitamin C-rich foods is limited. It is worth noting that our study only monitored offspring until adolescence, leaving the possibility that more differences could emerge as the animals age further. While further research is necessary to directly translate these findings to humans, our results nevertheless underscore the critical importance of maintaining adequate and consistent vitamin C intake during pregnancy to ensure positive outcomes for offspring.

## Figures and Tables

**Table 1:** Maternal physical characteristics.

Characteristics	Optimal	(*n*)	Low	(*n*)	Low-Optimal	(*n*)	Optimal-Low	(*n)*
Weight (g) at enrolment	451.9 ± 49.0	35	432.7 ± 48.1	38	439.7 ± 37.7	22	428.6 ± 46.0	22
Weight (g) at mating	632.7 ± 111.8	34	588.5 ± 85.9	36	579.2 ± 73.1	22	594.1 ± 85.8	22
Age (weeks) at mating	14.9 ± 4.0	34	16.1 ± 5.2	36	15.4 ± 4.7	22	15.2 ± 4.5	22

Data are presented as group means ± SD and were analysed using one-way ANOVA.

**Table 2:** Pregnancy outcomes.

Outcomes	Optimal	(*n*)	Low	(*n*)	Low-Optimal	(*n*)	Optimal-Low	(*n*)
Miscarriage (delivery before GA62) (*n* = total pregnancies)	4 (11.8%)	34	2 (5.6%)	36	0	22	1 (4.5%)	22
Foetal reabsorption (*n* = total pregnancies)	2 (5.9%)	34	9 (25%)	36	5 (22.7%)	22	3 (13.6%)	22
Premature delivery (born GA62–66) (*n* = litters born GA62+)	0	30	2 (5.9%)	34	2 (9.1%)	22	1 (4.8%)	21
Stillbirth # of pregnancies (*n* = litters born GA62+)	5 (16.7%)	30	9 (26.5%)	34	1 (4.5%)	22	2 (9.5%)	21
Cumulative adverse outcome (*n* = total pregnancies)	11 (32.4%)	34	22 (61.1%)	36	7 (31.8%)	22	6 (27.3%) *	22
Stillbirth # of pups (*n* = total pups)	6 (5.9%)	102	11 (13.1%)	85	2 (3.9%)	51	1 (1.6%) *	61
Litter size (*n* pups) (*n* = term litters (born GA66+))	3.4 ± 1.0 (2–6)	30	2.6 ± 0.9 (1–4)	32	2.2 ± 0.8 (1–4) *	20	2.9 ± 1.0 (1–4)	20
GA of pups at delivery (*n* = term litters of 2–4 pups) †	68.6 ± 1.0	27	68.9 ± 1.2	27	69.5 ± 1.3	17	69.2 ± 1.0	18
Litter birth weight (g) (*n* = term litters of 2–4 pups) †	95.3 ± 10.4	27	97.2 ± 10.0	27	105.5 ± 11.7 *	17	87.6 ± 11.4	18
Pup sexes (*n* = total liveborn pups)	49♂ 47♀	96	37♂ 37♀	74	24♂ 25♀	49	30♂ 30♀	60

Rates of miscarriage, foetal reabsorption, premature delivery, stillbirth (# of pregnancies and # of pups), and the cumulative adverse outcome are presented as absolute counts with the proportion of the total shown in brackets. Litter size is presented as group means ± SD with smallest to largest litter sizes shown in brackets. GA of pups at delivery and litter birth weight are presented as group means ± SD. Data were analysed using one-way ANOVA or Fisher’s exact test to compare proportions. * denotes significance of *p* < 0.05. † Three low-optimal litters of 1, two low-optimal premature litters, two optimal-low litters of 1, and one optimal-low premature litter were excluded from the analysis of GA of pups at delivery and litter birth weight.

**Table 3:** Body weights and measurements on day zero.

Characteristics	Males				Females			
	Optimal (*n* = 44)	Low (*n* = 36)	Low-Optimal (*n* = 24)	Optimal-Low (*n* = 30)	Optimal (*n* = 42)	Low (*n* = 35)	Low-Optimal (*n* = 25)	Optimal-Low (*n* = 30)
Body weight (g)	93.3 ± 14.8	96.3 ± 13.2	102.2 ± 13.3 *	87.8 ± 16.0	91.4 ± 12.9	93.5 ± 14.8	104.2 ± 13.1 *	86.5 ± 14.4
Crown-rump (mm)	128.8 ± 8.8	129.0 ± 9.0	135.5 ± 8.5 *	126.1 ± 10.8	127.7 ± 8.7	126.0 ± 8.7	137.5 ± 8.5 *	124.4 ± 8.9
Hind limb (mm)	38.5 ± 5.0	35.1 ± 3.7	37.0 ± 2.5	36.6 ± 3.8	36.7 ± 4.3	34.7 ± 3.6	36.6 ± 3.4	33.3 ± 4.5 *
Hock-toe (mm)	38.2 ± 5.1	36.8 ± 3.1	35.9 ± 2.3	36.6 ± 3.4	37.3 ± 4.6	35.0 ± 3.6	37.4 ± 3.0	34.7 ± 3.0
Ponderal Index (PI)	20.2 ± 3.4	22.3 ± 2.9	20.0 ± 1.8 *	20.5 ± 3.6	20.6 ± 3.3	23.2 ± 2.7	20.3 ± 2.2 *	22.7 ± 4.7

Data are presented as group means ± SD and were analysed within sex using one-way ANOVA. * denotes significance of *p* < 0.05. These data represent all newborn pups irrespective of later group allocation.

**Table 4:** Organ weights on day zero.

Characteristics	Males				Females			
	Optimal (*n* = 23)	Low (*n* = 19)	Low-Optimal (*n* = 6)	Optimal-Low (*n* = 12)	Optimal (*n* = 22)	Low (*n* = 18)	Low-Optimal (*n* = 9)	Optimal-Low (*n* = 9)
Brain-to-body wgt	2.67 ± 0.43	2.58 ± 0.29	2.65 ± 0.36	2.83 ± 0.35	2.71 ± 0.46	2.52 ± 0.30	2.42 ± 0.27	2.87 ± 0.42
Liver-to-body wgt	3.94 ± 0.74	3.79 ± 0.36	4.22 ± 0.37	3.34 ± 0.44 *	4.26 ± 0.56	3.88 ± 0.52	4.01 ± 0.73	3.70 ± 0.53
Brain-to-liver ratio	0.71 ± 0.19	0.68 ± 0.11	0.63 ± 0.09	0.86 ± 0.17 *	0.66 ± 0.16	0.67 ± 0.11	0.61 ± 0.08	0.80 ± 0.20
Heart-to-body wgt	0.41 ± 0.04	0.40 ± 0.05	0.44 ± 0.02	0.44 ± 0.05	0.43 ± 0.04	0.40 ± 0.03	0.43 ± 0.03	0.41 ± 0.05
Kidney-to-body wgt	0.43 ± 0.04	0.44 ± 0.04	0.44 ± 0.04	0.39 ± 0.04 *	0.43 ± 0.04	0.44 ± 0.04	0.42 ± 0.05	0.46 ± 0.04
Adrenal-to-body wgt	0.03 ± 0.02	0.02 ± 0.01	0.01 ± 0.004 *	0.01 ± 0.003 *	0.02 ± 0.01	0.05 ± 0.07	0.02 ± 0.004	0.03 ± 0.012
Testis-to-body wgt	0.07 ± 0.05	0.07 ± 0.09	0.04 ± 0.004 *	0.04 ± 0.004 *	N/A	N/A	N/A	N/A
Subcut. Fat-to-body wgt	1.50 ± 0.5	1.40 ± 0.3	1.20 ± 0.5	1.50 ± 0.2	1.34 ± 0.4	1.30 ± 0.3	1.38 ± 0.3	1.50 ± 0.2
Visc. Fat-to-body wgt	1.06 ± 0.3	1.04 ± 0.4	0.84 ± 0.12	1.05 ± 0.18	0.73 ± 0.3	0.81 ± 0.2	0.65 ± 0.08	0.81 ± 0.18

Data are presented as group means ± SD and were analysed within sex using one-way ANOVA. * denotes significance of *p* < 0.05. All weights are in grams and are expressed as percentages of body weight. Paired organ weights were averaged for each animal. Wgt = weight, subcut. = subcutaneous, visc. = visceral. These data derive from pups allocated at birth to the neonate group.

**Table 5:** Body measurements at weaning (Day 21).

Characteristics	Males				Females			
	Optimal (*n* = 21)	Low (*n* = 17)	Low-Optimal (*n* = 17)	Optimal-Low (*n* = 18)	Optimal (*n* = 20)	Low (*n* = 17)	Low-Optimal (*n* = 16)	Optimal-Low (*n* = 20)
Crown-rump (CRL)	171.9 ± 14.7	179.3 ± 17.8	169.0 ± 19.7	169.1 ± 11.7	171.5 ± 14.1	168.1 ± 14.1	174.5 ± 16.0	166.6 ± 15.9
CRL increase from birth	36.9 ± 16.3	47.7 ± 17.3	39.5 ± 14.8	44.5 ± 11.5	40.5 ± 13.6	42.2 ± 15.7	39.5 ± 13.0	44.2 ± 10.1
Hind limb (HL)	45.2 ± 6.3	45.3 ± 3.3	41.4 ± 2.8	44.0 ± 4.6	43.9 ± 4.0	43.6 ± 3.9	43.7 ± 6.1	41.8 ± 4.7
HL increase from birth	6.9 ± 5.9	9.6 ± 3.5	6.9 ± 3.6	6.2 ± 4.3	6.2 ± 3.6	9.4 ± 4.4	8.1 ± 4.0	9.0 ± 4.9
Hock-toe (HT)	40.3 ± 4.4	41.1 ± 5.3	39.4 ± 2.8	41.6 ± 5.1	40.1 ± 4.6	39.6 ± 3.5	40.7 ± 3.4	38.1 ± 3.3
HT increase from birth	3.8 ± 4.5	4.7 ± 4.6	3.6 ± 2.0	4.5 ± 3.2	2.5 ± 2.1	4.5 ± 2.6	3.9 ± 3.4	4.0 ± 2.0
Ponderal Index (PI)	20.5 ± 2.5	20.1 ± 2.6	21.2 ± 2.8	19.7 ± 3.0	21.5 ± 2.7	21.3 ± 2.5	22.5 ± 4.2	21.4 ± 3.6

Data are presented as group means ± SD and were analysed within sex using one-way ANOVA. All measurements are in millimetres. Data are pooled from pups randomised at birth to the juvenile and adolescent groups.

**Table 6:** Organ weights on day 28.

Characteristics	Males				Females			
	Optimal (*n* = 13)	Low (*n* = 10)	Low-Optimal (*n* = 9)	Optimal-Low (*n* = 10)	Optimal (*n* = 11)	Low (*n* = 10)	Low-Optimal (*n* = 8)	Optimal-Low (*n* = 11)
Brain-to-body wgt	1.31 ± 0.29	1.23 ± 0.20	1.57 ± 0.48	1.31 ± 0.14	1.30 ± 0.26	1.42 ± 0.27	1.32 ± 0.24	1.32 ± 0.19
Liver-to-body wgt	2.99 ± 0.45	3.35 ± 0.42	3.04 ± 0.32	3.01 ± 0.64	3.12 ± 0.68	3.11 ± 0.52	3.10 ± 0.33	3.24 ± 0.30
Heart-to-body wgt	0.33 ± 0.02	0.32 ± 0.03	0.30 ± 0.02	0.30 ± 0.03	0.30 ± 0.02	0.30 ± 0.04	0.28 ± 0.01	0.29 ± 0.02
Kidney-to-body wgt	0.42 ± 0.05	0.46 ± 0.08	0.47 ± 0.11	0.45 ± 0.04	0.43 ± 0.04	0.46 ± 0.03	0.37 ± 0.17	0.44 ± 0.03
Adrenal-to-body wgt	0.02 ± 0.01	0.02 ± 0.01	0.03 ± 0.01 *	0.02 ± 0.004	0.03 ± 0.01	0.03 ± 0.01	0.03 ± 0.01	0.03 ± 0.01
Testis-to-body wgt	0.14 ± 0.04	0.09 ± 0.03	0.09 ± 0.06	0.09 ± 0.02	N/A	N/A	N/A	N/A
Subcut. Fat-to-body wgt	0.64 ± 0.27	0.77 ± 0.28	0.60 ± 0.27	0.89 ± 0.23	0.83 ± 0.37	0.68 ± 0.25	0.82 ± 0.21	0.83 ± 0.24
Visc. Fat-to-body wgt	0.17 ± 0.10	0.28 ± 0.12	0.15 ± 0.15	0.19 ± 0.09	0.26 ± 0.20	0.16 ± 0.06	0.22 ± 0.11	0.15 ± 0.06

Data are presented as group means ± SD and were analysed within sex using one-way ANOVA. * denotes significance of *p* < 0.05. All weights are in grams and are expressed as percentages of body weight. Paired organ weights were averaged for each animal. Wgt = weight, subcut. = subcutaneous, visc. = visceral. These data derive from pups randomised at birth to the juvenile group.

**Table 7:** Organ weights at four months.

Characteristics	Males				Females			
	Optimal (*n* = 8)	Low (*n* = 7)	Low-Optimal (*n* = 8)	Optimal-Low (*n* = 8)	Optimal (*n* = 9)	Low (*n* = 7)	Low-Optimal (*n* = 8)	Optimal-Low (*n* = 9)
Brain-to-body wgt	0.49 ± 0.06	0.51 ± 0.04	0.52 ± 0.05	0.58 ± 0.03 *	0.53 ± 0.08	0.56 ± 0.07	0.54 ± 0.05	0.57 ± 0.08
Liver-to-body wgt	3.06 ± 0.33	2.95 ± 0.30	2.91 ± 0.32	3.21 ± 0.23	3.30 ± 0.40	3.23 ± 0.50	3.35 ± 0.26	3.35 ± 0.52
Heart-to-body wgt	0.26 ± 0.01	0.27 ± 0.01	0.25 ± 0.03	0.26 ± 0.03	0.26 ± 0.02	0.27 ± 0.03	0.26 ± 0.02	0.27 ± 0.02
Kidney-to-body wgt	0.29 ± 0.03	0.30 ± 0.02	0.30 ± 0.03	0.30 ± 0.03	0.28 ± 0.03	0.30 ± 0.03	0.30 ± 0.03	0.32 ± 0.02
Adrenal-to-body wgt	0.02 ± 0.004	0.02 ± 0.004	0.02 ± 0.003	0.02 ± 0.003	0.03 ± 0.004	0.02 ± 0.002	0.02 ± 0.002	0.03 ± 0.004
Testis-to-body wgt	0.24 ± 0.05	0.16 ± 0.14	0.22 ± 0.04	0.24 ± 0.04	N/A	N/A	N/A	N/A
Subcut. Fat-to-body wgt	0.77 ± 0.27	0.91 ± 0.22	0.86 ± 0.13	0.58 ± 0.11 *	1.03 ± 0.30	1.22 ± 0.44	1.25 ± 0.19	1.20 ± 0.16
Visc. Fat-to-body wgt	0.48 ± 0.17	0.53 ± 0.09	0.60 ± 0.11	0.43 ± 0.10 *	0.59 ± 0.18	0.53 ± 0.17	0.73 ± 0.12	0.59 ± 0.12

Data are presented as group means ± SD and were analysed within sex using one-way ANOVA. * denotes significance of *p* < 0.05. All weights are in grams and are expressed as percentages of body weight. Paired organ weights were averaged for each animal. Wgt = weight, subcut. = subcutaneous, visc. = visceral. These data derive from pups randomised at birth to the adolescent group.

**Table 8:** Glucose tolerance test characteristics.

Characteristics	Males				Females			
	Optimal (*n* = 8)	Low (*n* = 6)	Low-Optimal (*n* = 6)	Optimal-Low (*n* = 5)	Optimal (*n* = 8)	Low (*n* = 8)	Low-Optimal (*n* = 5)	Optimal-Low (*n* = 9)
Absolute peak (mmol/L)	14.7 ± 4.1	15.0 ± 2.0	17.6 ± 2.4	16.4 ± 2.1	13.3 ± 2.0	14.6 ± 2.1	16.1 ± 2.5	15.0 ± 2.7
Delta-peak (mmol/L)	6.6 ± 3.3	6.8 ± 1.9	9.7 ± 1.9	8.5 ± 2.2	5.1 ± 1.3	7.1 ± 2.6	7.1 ± 2.0	6.2 ± 2.6
Time to peak (min)	48.8 ± 15.5	55.0 ± 12.3	60.0 ± 0.0	54.0 ± 13.4	41.3 ± 15.5	52.5 ± 13.9	37.5 ± 15.0	40.0 ± 15.0
AUC (mmol/L × min)	1555 ± 608.1	1651 ± 1032	3101 ± 925.2 *	2061 ± 902.4	749.4 ± 295.6	1695 ± 1642	1569 ± 1016	1129 ± 709.1

Data are presented as group means ± SD and were analysed within sex using one-way ANOVA. * denotes significance of *p* < 0.05.

**Table 9:** Offspring mortality.

Maternal Diet	Sex	Age	Euthanised or Spontaneous Death	Euthanasia or Autopsy Notes
Low	Male	Two months	Euthanised	Weight loss, loss of appetite, scruffy appearance. Abnormal maxillary incisors growing in on themselves. Animal likely in pain and struggling to eat.
Low-optimal	Male	25 days	Spontaneous Death	Autopsy revealed enlarged heart and clotted blood in the heart and lungs. Assumed aspiration of foreign object.
Optimal-low	Female	Four days	Spontaneous Death	Runt of the litter, born small at 56.8 g, failed to regain birth weight during the first few days even with supplemental formula feeding.

## Data Availability

The original contributions presented in the study are included in the article/Supplementary Materials; further enquires can be directed to the corresponding author.

## Supplementary Materials

The following supporting information can be downloaded at: https://www.mdpi.com/article/10.3390/nu16030369/s1, Table S1: Maternal physical characteristics, Table S2: Pregnancy weight gain, Table S3: Pregnancy outcomes, Table S4: Maternal salivary cortisol concentrations, Table S5: Relative organ weights and body measurements at birth, Table S6: Fractional weight gain, Table S7: Body measurements at weaning, Table S8: Relative organ weights at 28 days of age, Table S9: Weight, growth rate from birth to four months, Table S10: Relative organ weights at four months of age, Table S11: Oral glucose tolerance tests, Table S12: Offspring salivary cortisol concentrations.

## References

[1] Meng R., Lv J., Yu C., Guo Y., Bian Z., Yang L., Chen Y., Zhang H., Chen X., Chen J. (2018). Prenatal famine exposure, adulthood obesity patterns and risk of type 2 diabetes. Int. J. Epidemiol..

[2] Lumey L.H., Khalangot M.D., Vaiserman A.M. (2015). Association between type 2 diabetes and prenatal exposure to the Ukraine famine of 1932–33: A retrospective cohort study. Lancet Diabetes Endocrinol..

[3] Smith E.V.L., Dyson R.M., Vanderboor C.M.G., Sarr O., Anderson J., Berry M.J., Regnault T.R.H., Peng L., Gray C. (2022). Maternal Fructose Intake Causes Developmental Reprogramming of Hepatic Mitochondrial Catalytic Activity and Lipid Metabolism in Weanling and Young Adult Offspring. Int. J. Mol. Sci..

[4] Bayol S.A., Simbi B.H., Fowkes R.C., Stickland N.C. (2010). A maternal “junk food” diet in pregnancy and lactation promotes nonalcoholic Fatty liver disease in rat offspring. Endocrinology.

[5] Roseboom T.J., van der Meulen J.H., Osmond C., Barker D.J., Ravelli A.C., Schroeder-Tanka J.M., van Montfrans G.A., Michels R.P., Bleker O.P. (2000). Coronary heart disease after prenatal exposure to the Dutch famine, 1944–1945. Heart.

[6] Ekamper P., van Poppel F., Stein A.D., Bijwaard G.E., Lumey L.H. (2015). Prenatal famine exposure and adult mortality from cancer, cardiovascular disease, and other causes through age 63 years. Am. J. Epidemiol..

[7] Ravelli A.C., van der Meulen J.H., Michels R.P., Osmond C., Barker D.J., Hales C.N., Bleker O.P. (1998). Glucose tolerance in adults after prenatal exposure to famine. Lancet.

[8] Ravelli A.C., van Der Meulen J.H., Osmond C., Barker D.J., Bleker O.P. (1999). Obesity at the age of 50 y in men and women exposed to famine prenatally. Am. J. Clin. Nutr..

[9] Drouin G., Godin J.R., Page B. (2011). The genetics of vitamin C loss in vertebrates. Curr. Genom..

[10] Rajan D.P., Huang W., Dutta B., Devoe L.D., Leibach F.H., Ganapathy V., Prasad P.D. (1999). Human placental sodium-dependent vitamin C transporter (SVCT2): Molecular cloning and transport function. Biochem. Biophys. Res. Commun..

[11] Sotiriou S., Gispert S., Cheng J., Wang Y., Chen A., Hoogstraten-Miller S., Miller G.F., Kwon O., Levine M., Guttentag S.H. (2002). Ascorbic-acid transporter Slc23a1 is essential for vitamin C transport into the brain and for perinatal survival. Nat. Med..

[12] Jain S.K., Wise R., Yanamandra K., Dhanireddy R., Bocchini J.A. (2008). The effect of maternal and cord-blood vitamin C, vitamin E and lipid peroxide levels on newborn birth weight. Mol. Cell. Biochem..

[13] Lee B.E., Hong Y.C., Lee K.H., Kim Y.J., Kim W.K., Chang N.S., Park E.A., Park H.S., Hann H.J. (2004). Influence of maternal serum levels of vitamins C and E during the second trimester on birth weight and length. Eur. J. Clin. Nutr..

[14] Jang W., Kim H., Lee B.E., Chang N. (2018). Maternal fruit and vegetable or vitamin C consumption during pregnancy is associated with fetal growth and infant growth up to 6 months: Results from the Korean Mothers and Children’s Environmental Health (MOCEH) cohort study. Nutr. J..

[15] Kishimoto Y., Kanai T., Sato K., Lee J., Jeong K.S., Shimokado K., Maruyama N., Ishigami A. (2013). Insufficient ascorbic acid intake during gestation induces abnormal cardiac dilation in fetal and neonatal SMP30/GNL knockout mice. Pediatr. Res..

[16] Schjoldager J.G., Paidi M.D., Lindblad M.M., Birck M.M., Kjærgaard A.B., Dantzer V., Lykkesfeldt J., Tveden-Nyborg P. (2015). Maternal vitamin C deficiency during pregnancy results in transient fetal and placental growth retardation in guinea pigs. Eur. J. Nutr..

[17] Habibzadeh N., Schorah C.J., Smithells R.W. (1986). The effects of maternal folic acid and vitamin C nutrition in early pregnancy on reproductive performance in the guinea-pig. Br. J. Nutr..

[18] Tveden-Nyborg P., Vogt L., Schjoldager J.G., Jeannet N., Hasselholt S., Paidi M.D., Christen S., Lykkesfeldt J. (2012). Maternal vitamin C deficiency during pregnancy persistently impairs hippocampal neurogenesis in offspring of guinea pigs. PLoS ONE.

[19] Tveden-Nyborg P., Johansen L.K., Raida Z., Villumsen C.K., Larsen J.O., Lykkesfeldt J. (2009). Vitamin C deficiency in early postnatal life impairs spatial memory and reduces the number of hippocampal neurons in guinea pigs. Am. J. Clin. Nutr..

[20] Paidi M.D., Schjoldager J.G., Lykkesfeldt J., Tveden-Nyborg P. (2014). Prenatal vitamin C deficiency results in differential levels of oxidative stress during late gestation in foetal guinea pig brains. Redox Biol..

[21] Lykkesfeldt J., Trueba G.P., Poulsen H.E., Christen S. (2007). Vitamin C deficiency in weanling guinea pigs: Differential expression of oxidative stress and DNA repair in liver and brain. Br. J. Nutr..

[22] Harrison F.E., Dawes S.M., Meredith M.E., Babaev V.R., Li L., May J.M. (2010). Low vitamin C and increased oxidative stress and cell death in mice that lack the sodium-dependent vitamin C transporter SVCT2. Free Radic. Biol. Med..

[23] Liggins G.C. (1994). The role of cortisol in preparing the fetus for birth. Reprod. Fertil. Dev..

[24] Chadio S.E., Kotsampasi B., Papadomichelakis G., Deligeorgis S., Kalogiannis D., Menegatos I., Zervas G. (2007). Impact of maternal undernutrition on the hypothalamic-pituitary-adrenal axis responsiveness in sheep at different ages postnatal. J. Endocrinol..

[25] Kapoor A., Dunn E., Kostaki A., Andrews M.H., Matthews S.G. (2006). Fetal programming of hypothalamo-pituitary-adrenal function: Prenatal stress and glucocorticoids. J. Physiol..

[26] Kapoor A., Matthews S.G. (2005). Short periods of prenatal stress affect growth, behaviour and hypothalamo-pituitary-adrenal axis activity in male guinea pig offspring. J. Physiol..

[27] Shaw J.C., Palliser H.K., Dyson R.M., Hirst J.J., Berry M.J. (2016). Long-term effects of preterm birth on behavior and neurosteroid sensitivity in the guinea pig. Pediatr. Res..

[28] Bruehl H., Rueger M., Dziobek I., Sweat V., Tirsi A., Javier E., Arentoft A., Wolf O.T., Convit A. (2007). Hypothalamic-pituitary-adrenal axis dysregulation and memory impairments in type 2 diabetes. J. Clin. Endocrinol. Metab..

[29] Wirtz P.H., von Kanel R., Emini L., Ruedisueli K., Groessbauer S., Maercker A., Ehlert U. (2007). Evidence for altered hypothalamus-pituitary-adrenal axis functioning in systemic hypertension: Blunted cortisol response to awakening and lower negative feedback sensitivity. Psychoneuroendocrinology.

[30] Holsboer F. (2000). The corticosteroid receptor hypothesis of depression. Neuropsychopharmacology.

[31] Walker E.F., Trotman H.D., Pearce B.D., Addington J., Cadenhead K.S., Cornblatt B.A., Heinssen R., Mathalon D.H., Perkins D.O., Seidman L.J. (2013). Cortisol levels and risk for psychosis: Initial findings from the North American prodrome longitudinal study. Biol. Psychiatry.

[32] Xiong F., Zhang L. (2013). Role of the hypothalamic-pituitary-adrenal axis in developmental programming of health and disease. Front. Neuroendocrinol..

[33] Sheng J.A., Bales N.J., Myers S.A., Bautista A.I., Roueinfar M., Hale T.M., Handa R.J. (2020). The Hypothalamic-Pituitary-Adrenal Axis: Development, Programming Actions of Hormones, and Maternal-Fetal Interactions. Front. Behav. Neurosci..

[34] Coker S.J., Smith-Diaz C.C., Dyson R.M., Vissers M.C.M., Berry M.J. (2022). The Epigenetic Role of Vitamin C in Neurodevelopment. Int. J. Mol. Sci..

[35] Lykkesfeldt J., Tveden-Nyborg P. (2019). The Pharmacokinetics of Vitamin C. Nutrients.

[36] Coker S.J., Dyson R.M., Smith-Díaz C.C., Vissers M.C.M., Berry M.J. (2023). Effects of Low Vitamin C Intake on Fertility Parameters and Pregnancy Outcomes in Guinea Pigs. Nutrients.

[37] Kilkenny C., Browne W.J., Cuthill I.C., Emerson M., Altman D.G. (2012). Improving bioscience research reporting: The ARRIVE guidelines for reporting animal research. Osteoarthr. Cartil..

[38] Smith E.V.L., Dyson R.M., Berry M.J., Gray C. (2020). Fructose Consumption During Pregnancy Influences Milk Lipid Composition and Offspring Lipid Profiles in Guinea Pigs. Front. Endocrinol..

[39] Morrison J.L., Botting K.J., Darby J.R.T., David A.L., Dyson R.M., Gatford K.L., Gray C., Herrera E.A., Hirst J.J., Kim B. (2018). Guinea pig models for translation of the developmental origins of health and disease hypothesis into the clinic. J. Physiol..

[40] Martinho F. (2006). Dystocia caused by ectopic pregnancy in a guinea pig (*Cavia porcellus*). Vet. Clin. Exot. Anim. Pract..

[41] Nardozza L.M., Caetano A.C., Zamarian A.C., Mazzola J.B., Silva C.P., Marcal V.M., Lobo T.F., Peixoto A.B., Araujo Junior E. (2017). Fetal growth restriction: Current knowledge. Arch. Gynecol. Obstet..

[42] Mitchell M.L. (2001). Fetal brain to liver weight ratio as a measure of intrauterine growth retardation: Analysis of 182 stillborn autopsies. Mod. Pathol..

[43] Longo S., Borghesi A., Tzialla C., Stronati M. (2014). IUGR and infections. Early Hum. Dev..

[44] Nowakowska B.A., Pankiewicz K., Nowacka U., Niemiec M., Kozlowski S., Issat T. (2021). Genetic Background of Fetal Growth Restriction. Int. J. Mol. Sci..

[45] Lumey L.H. (1992). Decreased birthweights in infants after maternal in utero exposure to the Dutch famine of 1944–1945. Paediatr. Perinat. Epidemiol..

[46] Odegard R.A., Vatten L.J., Nilsen S.T., Salvesen K.A., Austgulen R. (2000). Preeclampsia and fetal growth. Obstet. Gynecol..

[47] Ibanez L., Suarez L., Lopez-Bermejo A., Diaz M., Valls C., de Zegher F. (2008). Early development of visceral fat excess after spontaneous catch-up growth in children with low birth weight. J. Clin. Endocrinol. Metab..

[48] Embleton N.D., Korada M., Wood C.L., Pearce M.S., Swamy R., Cheetham T.D. (2016). Catch-up growth and metabolic outcomes in adolescents born preterm. Arch. Dis. Child..

[49] Singhal A. (2015). Should We Promote Catch-Up Growth or Growth Acceleration in Low-Birthweight Infants?. Nestle Nutr. Inst. Workshop Ser..

[50] Mace K., Shahkhalili Y., Aprikian O., Stan S. (2006). Dietary fat and fat types as early determinants of childhood obesity: A reappraisal. Int. J. Obes..

[51] Carberry A.E., Colditz P.B., Lingwood B.E. (2010). Body composition from birth to 4.5 months in infants born to non-obese women. Pediatr. Res..

[52] Gungor D.E., Paul I.M., Birch L.L., Bartok C.J. (2010). Risky vs. rapid growth in infancy: Refining pediatric screening for childhood overweight. Arch. Pediatr. Adolesc. Med..

[53] Wu G., Bazer F.W., Cudd T.A., Meininger C.J., Spencer T.E. (2004). Maternal nutrition and fetal development. J. Nutr..

[54] Zhang D.L., Du Q., Djemli A., Julien P., Fraser W.D., Luo Z.C. (2017). Early and Late Postnatal Accelerated Growth Have Distinct Effects on Metabolic Health in Normal Birth Weight Infants. Front. Endocrinol..

[55] Metcalfe N.B., Monaghan P. (2001). Compensation for a bad start: Grow now, pay later?. Trends Ecol. Evol..

[56] Buckberry S., Bianco-Miotto T., Bent S.J., Dekker G.A., Roberts C.T. (2014). Integrative transcriptome meta-analysis reveals widespread sex-biased gene expression at the human fetal-maternal interface. Mol. Hum. Reprod..

[57] Broere-Brown Z.A., Baan E., Schalekamp-Timmermans S., Verburg B.O., Jaddoe V.W., Steegers E.A. (2016). Sex-specific differences in fetal and infant growth patterns: A prospective population-based cohort study. Biol. Sex Differ..

[58] Eriksson J.G., Kajantie E., Osmond C., Thornburg K., Barker D.J. (2010). Boys live dangerously in the womb. Am. J. Hum. Biol..

[59] Kraemer S. (2000). The fragile male. BMJ.

[60] Renaville R., Hammadi M., Portetelle D. (2002). Role of the somatotropic axis in the mammalian metabolism. Domest. Anim. Endocrinol..

[61] Hoffman M.L., Reed S.A., Pillai S.M., Jones A.K., McFadden K.K., Zinn S.A., Govoni K.E. (2017). Physiology and endocrinology symposium: The effects of poor maternal nutrition during gestation on offspring postnatal growth and metabolism. J. Anim. Sci..

[62] Bauer M.K., Breier B.H., Harding J.E., Veldhuis J.D., Gluckman P.D. (1995). The fetal somatotropic axis during long term maternal undernutrition in sheep: Evidence for nutritional regulation in utero. Endocrinology.

[63] Rehfeldt C., Nissen P.M., Kuhn G., Vestergaard M., Ender K., Oksbjerg N. (2004). Effects of maternal nutrition and porcine growth hormone (pGH) treatment during gestation on endocrine and metabolic factors in sows, fetuses and pigs, skeletal muscle development, and postnatal growth. Domest. Anim. Endocrinol..

[64] Hoffman M.L., Rokosa M.A., Zinn S.A., Hoagland T.A., Govoni K.E. (2014). Poor maternal nutrition during gestation in sheep reduces circulating concentrations of insulin-like growth factor-I and insulin-like growth factor binding protein-3 in offspring. Domest. Anim. Endocrinol..

[65] Hoffman M.L., Peck K.N., Forella M.E., Fox A.R., Govoni K.E., Zinn S.A. (2016). The effects of poor maternal nutrition during gestation on postnatal growth and development of lambs. J. Anim. Sci..

[66] George L.A., Zhang L., Tuersunjiang N., Ma Y., Long N.M., Uthlaut A.B., Smith D.T., Nathanielsz P.W., Ford S.P. (2012). Early maternal undernutrition programs increased feed intake, altered glucose metabolism and insulin secretion, and liver function in aged female offspring. Am. J. Physiol. Regul. Integr. Comp. Physiol..

[67] Freake H.C., Govoni K.E., Guda K., Huang C., Zinn S.A. (2001). Actions and interactions of thyroid hormone and zinc status in growing rats. J. Nutr..

[68] Rausch M.I., Tripp M.W., Govoni K.E., Zang W., Webert W.J., Crooker B.A., Hoagland T.A., Zinn S.A. (2002). The influence of level of feeding on growth and serum insulin-like growth factor I and insulin-like growth factor-binding proteins in growing beef cattle supplemented with somatotropin. J. Anim. Sci..

[69] Claahsen-van der Grinten H.L., Speiser P.W., Ahmed S.F., Arlt W., Auchus R.J., Falhammar H., Fluck C.E., Guasti L., Huebner A., Kortmann B.B.M. (2022). Congenital Adrenal Hyperplasia-Current Insights in Pathophysiology, Diagnostics, and Management. Endocr. Rev..

[70] Sohaib S.A., Hanson J.A., Newell-Price J.D., Trainer P.J., Monson J.P., Grossman A.B., Besser G.M., Reznek R.H. (1999). CT appearance of the adrenal glands in adrenocorticotrophic hormone-dependent Cushing’s syndrome. AJR Am. J. Roentgenol..

[71] Vining R.F., McGinley R.A., Symons R.G. (1983). Hormones in saliva: Mode of entry and consequent implications for clinical interpretation. Clin. Chem..

[72] Spencer R.L., Deak T. (2017). A users guide to HPA axis research. Physiol. Behav..

[73] Falhammar H., Frisen L., Hirschberg A.L., Norrby C., Almqvist C., Nordenskjold A., Nordenstrom A. (2015). Increased Cardiovascular and Metabolic Morbidity in Patients with 21-Hydroxylase Deficiency: A Swedish Population-Based National Cohort Study. J. Clin. Endocrinol. Metab..

[74] Kim M.S., Fraga N.R., Minaeian N., Geffner M.E. (2022). Components of Metabolic Syndrome in Youth with Classical Congenital Adrenal Hyperplasia. Front. Endocrinol..

[75] Scaroni C., Zilio M., Foti M., Boscaro M. (2017). Glucose Metabolism Abnormalities in Cushing Syndrome: From Molecular Basis to Clinical Management. Endocr. Rev..

[76] Horton D.M., Saint D.A., Owens J.A., Gatford K.L., Kind K.L. (2017). Use of the hyperinsulinemic euglycemic clamp to assess insulin sensitivity in guinea pigs: Dose response, partitioned glucose metabolism, and species comparisons. Am. J. Physiol. Regul. Integr. Comp. Physiol..

[77] Podell B.K., Ackart D.F., Richardson M.A., DiLisio J.E., Pulford B., Basaraba R.J. (2017). A model of type 2 diabetes in the guinea pig using sequential diet-induced glucose intolerance and streptozotocin treatment. Dis. Model. Mech..

[78] Kind K.L., Clifton P.M., Grant P.A., Owens P.C., Sohlstrom A., Roberts C.T., Robinson J.S., Owens J.A. (2003). Effect of maternal feed restriction during pregnancy on glucose tolerance in the adult guinea pig. Am. J. Physiol. Regul. Integr. Comp. Physiol..

[79] Ozanne S.E., Hales C.N. (2004). Lifespan: Catch-up growth and obesity in male mice. Nature.

[80] Jimenez-Chillaron J.C., Patti M.E. (2007). To catch up or not to catch up: Is this the question? Lessons from animal models. Curr. Opin. Endocrinol. Diabetes Obes..

[81] Ong K.K., Loos R.J. (2006). Rapid infancy weight gain and subsequent obesity: Systematic reviews and hopeful suggestions. Acta Paediatr..

[82] Demerath E.W., Reed D., Choh A.C., Soloway L., Lee M., Czerwinski S.A., Chumlea W.C., Siervogel R.M., Towne B. (2009). Rapid postnatal weight gain and visceral adiposity in adulthood: The Fels Longitudinal Study. Obesity.

[83] Van Hulst A., Barnett T.A., Paradis G., Roy-Gagnon M.H., Gomez-Lopez L., Henderson M. (2017). Birth Weight, Postnatal Weight Gain, and Childhood Adiposity in Relation to Lipid Profile and Blood Pressure during Early Adolescence. J. Am. Heart. Assoc..

[84] Fabricius-Bjerre S., Jensen R.B., Faerch K., Larsen T., Molgaard C., Michaelsen K.F., Vaag A., Greisen G. (2011). Impact of birth weight and early infant weight gain on insulin resistance and associated cardiovascular risk factors in adolescence. PLoS ONE.

[85] Godfrey K.M., Lillycrop K.A., Burdge G.C., Gluckman P.D., Hanson M.A. (2007). Epigenetic mechanisms and the mismatch concept of the developmental origins of health and disease. Pediatr. Res..

[86] Gluckman P.D., Hanson M.A., Low F.M. (2011). The role of developmental plasticity and epigenetics in human health. Birth Defects Res. Part C Embryo Today.

[87] Padayatty S.J., Levine M. (2016). Vitamin C: The known and the unknown and Goldilocks. Oral Dis..

[88] Tomas E., Lin Y.S., Dagher Z., Saha A., Luo Z., Ido Y., Ruderman N.B. (2002). Hyperglycemia and insulin resistance: Possible mechanisms. Ann. N. Y. Acad. Sci..

[89] Jellinger P.S. (2007). Metabolic consequences of hyperglycemia and insulin resistance. Clin. Cornerstone.

[90] Heijmans B.T., Tobi E.W., Stein A.D., Putter H., Blauw G.J., Susser E.S., Slagboom P.E., Lumey L.H. (2008). Persistent epigenetic differences associated with prenatal exposure to famine in humans. Proc. Natl. Acad. Sci. USA.

[91] Chen P.Y., Ganguly A., Rubbi L., Orozco L.D., Morselli M., Ashraf D., Jaroszewicz A., Feng S., Jacobsen S.E., Nakano A. (2013). Intrauterine calorie restriction affects placental DNA methylation and gene expression. Physiol. Genom..

[92] Blaschke K., Ebata K.T., Karimi M.M., Zepeda-Martinez J.A., Goyal P., Mahapatra S., Tam A., Laird D.J., Hirst M., Rao A. (2013). Vitamin C induces Tet-dependent DNA demethylation and a blastocyst-like state in ES cells. Nature.

[93] Yin R., Mao S.Q., Zhao B., Chong Z., Yang Y., Zhao C., Zhang D., Huang H., Gao J., Li Z. (2013). Ascorbic acid enhances Tet-mediated 5-methylcytosine oxidation and promotes DNA demethylation in mammals. J. Am. Chem. Soc..

