# Supplementary material for: Maternal Vitamin C Intake during Pregnancy Influences Long-Term Offspring Growth with Timing- and Sex-Specific Effects in Guinea Pigs
Source: Nutrients. 2024 Jan 26;16(3):369. doi: 10.3390/nu16030369 (PMC10857109; doi:10.3390/nu16030369)
Supplement: Supplementary file 1 [file nutrients-16-00369-s001.zip › nutrients-2806345-supplementary.pdf]

Table S1. Maternal physical characteristics (raw means and SD)

	**Optimal**	**Low**	**Low-optimal**	**Optimal-low**	**Model p-value**
Weight at enrolment (g)	451.9 ± 49.02 (*n* = 35)	432.7 ± 48.09 (*n* = 38)	439.7 ± 37.72 (*n* = 22)	428.6 ± 46.0 (*n* = 22)	0.2150
Weight at mating (g)	632.7 ± 111.8 (*n* = 34)	588.5 ± 85.86 (*n* = 36)	579.2 ± 73.12 (*n* = 22)	594.1 ± 85.77 (*n* = 22)	0.1165
Age at mating (weeks)	14.93 ± 4.019 (*n* = 34)	16.07 ± 5.185 (*n* = 36)	15.36 ± 4.658 (*n* = 22)	15.23 ± 4.466 (*n* = 22)	0.9188

Table S2. Pregnancy weight gain (g) (raw means and SD)

	**Optimal (*n* = 30)**	**Low (*n* = 32)**	**Low-optimal (*n* = 22)**	**Optimal-low (*n* = 21)**	**Model p-value**
Gestation week 0 = day of mating	638.96 ± 110.089	588.794 ± 83.849	579.168 ± 73.116	599.214 ± 84.405	0.0038*
Gestation week 1	662.887 ± 103.583	616.559 ± 86.763	599.682 ± 80.456	637.910 ± 79.384	
Gestation week 2	696.559 ± 103.179	646.019 ± 85.268	621.171 ± 84.027	667.676 ± 80.863	
Gestation week 3	723.630 ± 100.885	667.869 ± 83.064	647.336 ± 85.045	690.171 ± 72.528	
Gestation week 4	748.630 ± 96.957	689.613 ± 84.812	670.800 ± 81.239	712.510 ± 74.760	
Gestation week 5	800.287 ± 101.701	732.863 ± 85.484	726.423 ± 83.998	751.600 ± 80.034	
Gestation week 6	868.637 ± 112.673	786.025 ± 96.625	794.473 ± 91.471	809.862 ± 90.592	
Gestation week 7	958.543 ± 132.893	849.766 ± 112.145	852.673 ± 97.792	878.505 ± 99.409	
Gestation week 8	1014.153 ± 145.014	902.003 ± 115.823	914.162 ± 105.113	924.510 ± 99.561	
Gestation week 9 = week of delivery	1087.813 ± 163.763	944.772 ± 132.097	955.177 ± 105.285	964.562 ± 102.760	

Table S3. Pregnancy outcomes (raw means and SD)

	**Optimal**	**Low**	**Low-optimal**	**Optimal-low**	**p-values**
Miscarriage<br>(*n* = total pregnancies)	4 out of 34 = 11.765%	2 out of 36 = 5.556%	0 out of 22 = 0.000%	1 out of 22 = 4.545%	Low-optimal vs. optimal-low: >0.9999<br>Low-optimal vs. low: 0.5209<br>Low-optimal vs. optimal: 0.1462<br>Optimal-low vs. low: >0.9999<br>Optimal-low vs. optimal: 0.6381
Foetal reabsorption<br>(*n* = total pregnancies)	2 out of 34 = 5.882%	9 out of 36 = 25.000%	5 out of 22 = 22.727%	3 out of 22 = 13.636%	Low-optimal vs. optimal-low: 0.6981<br>Low-optimal vs. low: >0.9999<br>Low-optimal vs. optimal: 0.0865<br>Optimal-low vs. low: 0.4679<br>Optimal-low vs. optimal: 0.5843
Premature delivery<br>(*n* = litters born GA62+)	0 out 30 = 0.000%	2 out of 34 = 5.882%	2 out of 22 = 9.091%	1 out of 21 = 4.762%	Low-optimal vs. optimal-low: >0.9999<br>Low-optimal vs. low: 0.6416

					Low-optimal vs. optimal: 0.1500<br>Optimal-low vs. low: >0.9999<br>Optimal-low vs. optimal: 0.4118
Stillbirth # of pregnancies<br>(*n* = litters born GA62+)	5 out of 30 = 16.667%	9 out of 34 = 26.471%	1 out of 22 = 4.545%	2 out of 21 = 9.524%	Low-optimal vs. optimal-low: 0.6069<br>Low-optimal vs. low: 0.0703<br>Low-optimal vs. optimal: 0.2165<br>Optimal-low vs. low: 0.1742<br>Optimal-low vs. optimal: 0.4448
Cumulative adverse outcome<br>(*n* = total pregnancies)	11 out of 34 = 32.353%	22 out of 36 = 61.111%	7 out of 22 = 31.818%	6 out of 22 = 27.273%	Low-optimal vs. optimal-low: >0.9999<br>Low-optimal vs. low: 0.0570<br>Low-optimal vs. optimal: >0.9999<br>Optimal-low vs. low: 0.0160*<br>Optimal-low vs. optimal: 0.7718
Stillbirth # of pups<br>(*n* = total pups)	6 out of 102 = 5.882%	11 out of 85 = 13.095%	2 out of 51 = 3.922%	1 out of 61 = 1.639%	Low-optimal vs. optimal-low: 0.5905<br>Low-optimal vs. low: 0.1303<br>Low-optimal vs. optimal: 0.7195<br>Optimal-low vs. low: 0.0141*<br>Optimal-low vs. optimal: 0.2580
					**Model p-value**
Litter size	3.367 ± 0.9994 (*n* = 30)	2.625 ± 0.9070 (*n* = 32)	2.200 ± 0.7678 (*n* = 20)	2.900 ± 0.9679 (*n* = 20)	0.0002*
GA of pups at delivery	68.59 ± 1.047 (*n* = 27)	68.85 ± 1.231 (*n* = 27)	69.53 ± 1.328 (*n* = 17)	69.22 ± 1.003 (*n* = 18)	0.0525
Litter birth weight	95.32 ± 10.41 (*n* = 27)	97.16 ± 10.01 (*n* = 27)	105.5 ± 11.72 (*n* = 17)	85.57 ± 11.43 (*n* = 18)	<0.0001*

Table S4. Maternal salivary cortisol concentrations (ng/mL) (raw means and SD)

	**Optimal (*n* = 10)**	**Low (*n* = 10)**	**Low-optimal (*n* = 7)**	**Optimal-low (*n* = 7)**	**Model p-value**
Cortisol concentration	106.2 ± 69.26	87.56 ± 62.02	59.21 ± 47.55	58.78 ± 30.81	0.2636

Table S5. Relative organ weights and body measurements at birth (raw means and SD)

**Sex**	**Males**					**Females**				
**Maternal diet**	**Optimal (*n* = 23)**	**Low (*n* = 19)**	**Low-optimal (*n* = 6)**	**Optimal-low (*n* = 12)**	**Model p-value (male)**	**Optimal (*n* = 22)**	**Low (*n* = 18)**	**Low-optimal (*n* = 9)**	**Optimal-low (*n* = 9)**	**Model p-value (female)**
Body wgt (g)	93.33 ± 14.76	96.25 ± 13.21	102.2 ± 13.31	87.78 ± 15.99	0.0052*	91.36 ± 12.87	93.45 ± 14.78	104.2 ± 13.14	86.50 ± 14.40	<0.0001*
Brain wgt	2.672 ± 0.4328	2.581 ± 0.2935	2.652 ± 0.3603	2.827 ± 0.3475	0.2059	2.707 ± 0.4560	2.518 ± 0.2969	2.421 ± 0.2656	2.866 ± 0.4158	0.0423*

Liver wgt	3.936 ± 0.7353	3.786 ± 0.3614	4.220 ± 0.3656	3.336 ± 0.4352	0.0066*	4.255 ± 0.5623	3.877 ± 0.5245	4.007 ± 0.7255	3.702 ± 0.5326	0.0886
Brain-to-liver ratio	0.7073 ± 0.1885	0.6824 ± 0.1132	0.6311 ± 0.08915	0.8640 ± 0.1692	0.0070*	0.6560 ± 0.1640	0.6678 ± 0.1134	0.6141 ± 0.07761	0.8027 ± 0.2022	0.1292
Heart wgt	0.4148 ± 0.03946	0.4031 ± 0.04820	0.4437 ± 0.01855	0.4375 ± 0.05386	0.0995	0.4338 ± 0.04031	0.4030 ± 0.03486	0.4282 ± 0.03034	0.4145 ± 0.05319	0.0867
Kidney wgt	0.4253 ± 0.04484	0.4360 ± 0.03594	0.4426 ± 0.04142	0.3853 ± 0.03913	0.0400*	0.4338 ± 0.04267	0.4407 ± 0.04137	0.4199 ± 0.05236	0.4619 ± 0.03503	0.2197
Adrenal wgt	0.03094 ± 0.02154	0.02228 ± 0.01041	0.01327 ± 0.004354	0.01480 ± 0.003067	0.0007*	0.02407 ± 0.01431	0.04991 ± 0.07398	0.01899 ± 0.004208	0.02831 ± 0.01241	0.1593
Testis wgt	0.07463 ± 0.04613	0.07426 ± 0.09005	0.03571 ± 0.00369	0.03891 ± 0.00404	0.0022*	-	-	-	-	-
Subcut. Fat wgt	1.463 ± 0.4760	1.397 ± 0.3269	1.199 ± 0.4542	1.494 ± 0.1889	0.4303	1.342 ± 0.4003	1.297 ± 0.2676	1.378 ± 0.3190	1.495 ± 0.1968	0.4995
Visc. Fat wgt	1.059 ± 0.2720	1.040 ± 0.3524	0.8366 ± 0.1185	1.046 ± 0.1794	0.2273	0.7268 ± 0.2826	0.8140 ± 0.1664	0.6544 ± 0.08190	0.8148 ± 0.1840	0.2490
Crown rump (mm)	128.8 ± 8.819	129.0 ± 8.962	135.5 ± 8.487	126.1 ± 10.78	0.0124*	127.7 ± 8.695	126.0 ± 8.674	137.5 ± 8.478	124.4 ± 8.854	<0.0001*
Hind limb (mm)	38.48 ± 5.038	35.09 ± 3.675	37.00 ± 2.490	36.55 ± 3.841	0.0100*	36.74 ± 4.313	34.65 ± 3.611	36.55 ± 3.363	33.28 ± 4.479	0.0036*
Hock toe (mm)	38.18 ± 5.108	36.81 ± 3.105	35.90 ± 2.322	36.62 ± 2.296	0.4741	37.32 ± 4.614	35.0 ± 3.55	37.41 ± 3.034	34.69 ± 2.989	0.0176*
Ponderal index (kg/m^3)	20.16 ± 3.399	22.26 ± 2.923	20.02 ± 1.749	20.52 ± 3.368	0.0113*	20.64 ± 3.256	23.24 ± 2.698	20.30 ± 2.185	22.68 ± 4.655	0.0011*

All organ weights are in grams and are expressed as a percentage of body weight (g) on the day of euthanasia. For the female brain and hock toe, the p-value of < 0.05 did not remain significant after applying corrections for multiple comparisons.

Table S6. Fractional weight gain (mg/g per day) (adjusted means and SD)

**Sex**	**Maternal diet**	Day 1	Day 2	Day 3	Day 4	Day 5	Day 6	Day 7
**Male**	**Optimal (*n* = 21)**	-34.329 ± 30.274	-9.232 ± 31.049	8.569 ± 27.223	20.762 ± 24.696	24.485 ± 23.621	29.240 ± 24.512	35.629 ± 13.614
	**Low (*n* = 17)**	-55.411 ± 24.254	-19.907 ± 22.397	5.938 ± 21.318	17.234 ± 21.007	31.138 ± 20.682	28.428 ± 21.425	34.082 ± 17.822
	**Low-optimal (*n* = 17)**	-46.108 ± 28.457	-9.449 ± 27.417	8.259 ± 24.325	22.040 ± 23.447	27.688 ± 21.817	30.608 ± 21.637	31.913 ± 17.619
	**Optimal-low (*n* = 18)**	-39.528 ± 53.243	-19.944 ± 43.278	6.572 ± 33.405	11.472 ± 27.944	19.098 ± 27.166	32.137 ± 37.862	28.151 ± 18.971
**Model p-value (male)**		0.8623						
**Female**	**Optimal (*n* = 20)**	-39.781 ± 25.813	-2.923 ± 26.233	9.443 ± 26.382	22.001 ± 20.558	28.838 ± 24.086	28.614 ± 22.219	40.321 ± 18.081
	**Low (*n* = 17)**	-66.150 ± 24.681	-33.762 ± 19.032	-11.985 ± 17.945	5.471 ± 17.319	17.773 ± 18.864	20.597 ± 21.134	24.089 ± 17.221
	**Low-optimal (*n* = 16)**	-29.413 ± 45.015	5.195 ± 31.016	26.735 ± 25.700	43.950 ± 27.171	48.133 ± 26.002	49.447 ± 23.023	42.722 ± 21.079
	**Optimal-low (*n* = 20)**	-43.288 ± 27.846	-19.328 ± 24.846	-0.791 ± 23.262	10.232 ± 20.610	13.225 ± 12.239	25.084 ±16.829	25.352 ± 12.150
**Model p-value (female)**		0.0011*						

Table S7. Body measurements at weaning (raw means and SD)

Sex	Maternal diet	Crown rump (mm)	Hind limb (mm)	Hock toe (mm)	Ponderal index (kg/m^3)
**Male**	**Optimal (*n* = 21)**	171.9 ± 14.72	45.17 ± 6.261	40.28 ± 4.403	20.54 ± 2.502
	**Low (*n* = 17)**	179.3 ± 17.78	45.31 ± 3.301	41.08 ± 5.299	20.15 ± 2.578
	**Low-optimal (*n* = 17)**	169.0 ± 19.66	41.38 ± 2.754	39.44 ± 2.804	21.19 ± 2.777
	**Optimal-low (*n* = 18)**	169.1 ± 11.72	44.0 ± 4.604	41.63 ± 5.110	19.67 ± 2.927
**Model p-value (male)**		0.2992	0.0676	0.3833	0.4475
**Female**	**Optimal (*n* = 20)**	171.5 ± 14.06	43.88 ± 3.998	40.06 ± 4.630	21.54 ± 2.696
	**Low (*n* = 17)**	168.1 ± 14.11	43.64 ± 3.934	39.57 ± 2.804	21.27 ± 2.460
	**Low-optimal (*n* = 16)**	174.5 ± 16.03	43.73 ± 6.112	40.67 ± 3.416	22.51 ± 4.207
	**Optimal-low (*n* = 20)**	166.6 ± 15.91	41.82 ± 4.747	38.12 ± 3.276	21.44 ± 3.566
**Model p-value (female)**		0.4619	0.5616	0.2645	0.9980

Table S8. Relative organ weights at 28 days of age (adjusted means and SD)

Sex	Maternal diet	Brain wgt	Liver wgt	Heart wgt	Kidney wgt	Adrenal wgt	Testis wgt	Subcut. wgt	Visc. wgt
**Male**	**Optimal (*n* = 13)**	1.134 ± 0.2874	2.989 ± 0.4544	0.3263 ± 0.0247	0.4235 ± 0.0530	0.02006 ± 0.0069	0.1407 ± 0.0437	0.6431 ± 0.2746	0.1710 ± 0.0996
	**Low (*n* = 10)**	1.230 ± 0.2008	3.351 ± 0.4236	0.3218 ± 0.0294	0.4627 ± 0.0777	0.01976 ± 0.0057	0.09433 ± 0.0289	0.6010 ± 0.2679	0.1416 ± 0.1452
	**Low-optimal (*n* = 9)**	1.566 ± 0.4777	3.042 ± 0.3166	0.2993 ± 0.0240	0.4736 ± 0.1074	0.02824 ± 0.0089	0.08533 ± 0.0574	0.6010 ± 0.2679	0.1416 ± 0.1452
	**Optimal-low (*n* = 10)**	1.314 ± 0.1376	3.010 ± 0.6399	0.3038 ± 0.0254	0.4515 ± 0.0384	0.02174 ± 0.0036	0.08855 ± 0.0241	0.8854 ± 0.2303	0.1929 ± 0.0894
**Model p-value (male)**		0.2429	0.4631	0.0551	0.1652	0.0250*	0.1440	0.1016	0.0652
**Female**	**Optimal (*n* = 11)**	1.303 ± 0.2645	3.106 ± 0.6761	0.3015 ± 0.0206	0.4284 ± 0.0362	0.02607 ± 0.0116	-	0.8281 ± 0.3679	0.2552 ± 0.1977
	**Low (*n* = 10)**	1.420 ± 0.2718	3.111 ± 0.5250	0.3032 ± 0.0365	0.4573 ± 0.0333	0.02576 ± 0.0064	-	0.6754 ± 0.2472	0.1579 ± 0.0642
	**Low-optimal (*n* = 8)**	1.318 ± 0.2381	3.235 ± 0.2997	0.2946 ± 0.0218	0.4397 ± 0.0317	0.02744 ± 0.0076	-	0.8253 ± 0.2398	0.1523 ± 0.0641
	**Optimal-low (*n* = 11)**	1.319 ± 0.1850	3.235 ± 0.2997	0.2946 ± 0.0218	0.4397 ± 0.0317	0.02744 ± 0.0076	-	0.8253 ± 0.2398	0.1523 ± 0.0641
**Model p-value (female)**		0.6820	0.8901	0.2415	0.1900	0.3487	-	0.5476	0.1979

All organ weights are in grams and are expressed as a percentage of body weight (g) on the day of euthanasia.

Table S9. Weight, growth rate from birth to four months (adjusted means and SD)

**Males**	**Optimal (*n* = 21)**	**Low (*n* = 17)**	**Low-optimal (*n* = 17)**	**Optimal-low (*n* = 18)**	**Model p-value (male)**
Birth	94.257 ± 13.994	98.288 ± 13.897	96.729 ± 19.193	85.435 ± 16.470	< 0.0001*
Week 1	117.929 ± 21.316	122.635 ± 22.451	118.771 ± 29.908	100.606 ± 15.552	
Week 2	161.338 ± 33.802	174.09 ± 32.277	162.565 ± 41.344	142.976 ± 19.710	
Week 3	215.833 ± 45.884	226.712 ± 44.598	204.282 ± 54.308	187.182 ± 23.973	
Week 4	246.048 ± 49.404	259.588 ± 48.709	224.912 ± 71.030	217.176 ± 30.913	
Week 5	285.813 ± 68.498	308.171 ± 51.507	312.163 ± 70.356	227.729 ± 21.266	
Week 6	327.738 ± 73.246	358.543 ± 46.948	355.000 ± 82.184	270.929 ± 25.367	

Week 7	381.975 ± 78.439	411.971 ± 40.484	397.163 ± 90.286	310.686 ± 35.135	
Week 8	427.375 ± 73.568	452.629 ± 44.520	448.400 ± 90.420	355.886 ± 40.255	
Week 9	468.075 ± 88.704	491.486 ± 40.735	491.925 ± 92.527	386.214 ± 32.220	
Week 10	514.163 ± 88.253	532.300 ± 38.281	539.363 ± 99.915	419.229 ± 27.510	
Week 11	551.825 ± 102.909	569.071 ± 45.021	573.188 ± 98.856	453.486 ± 35.133	
Week 12	599.613 ± 106.369	605.457 ± 44.260	626.275 ± 97.353	488.800 ± 51.556	
Week 13	631.363 ± 115.569	646.686 ± 44.358	664.313 ± 88.468	523.729 ± 34.741	
Week 14	675.800 ± 117.569	676.714 ± 45.181	693.725 ± 87.213	556.586 ± 41.948	
Week 15	715.738 ± 112.300	707.629 ± 49.862	737.763 ± 78.557	596.471 ± 47.207	
Week 16	757.100 ± 107.071	725.743 ± 48.729	777.425 ± 80.282	631.271 ± 38.592	

**Females**	**Optimal (*n* = 20)**	**Low (*n* = 17)**	**Low-optimal (*n* =16)**	**Optimal-low (*n* = 20)**	**Model p-value (female)**
Birth	93.130 ± 10.600	93.382 ± 14.557	101.994 ± 14.495	88.805 ± 13.849	0.0143*
Week 1	117.833 ± 26.074	113.317 ± 23.062	138.919 ± 28.195	105.785 ± 21.642	
Week 2	162.338 ± 40.276	155.406 ± 35.024	185.725 ± 40.313	147.300 ± 29.556	
Week 3	212.214 ± 54.034	205.178 ± 45.618	239.006 ± 53.785	198.870 ± 39.991	
Week 4	243.519 ± 62.486	232.456 ± 53.232	269.156 ± 58.447	235.380 ± 51.183	
Week 5	296.167 ± 68.459	287.200 ± 75.494	350.663 ± 33.288	279.744 ± 59.782	
Week 6	341.244 ± 80.622	332.700 ± 75.531	382.500 ± 38.892	319.967 ± 65.066	
Week 7	391.189 ± 86.455	375.900 ± 79.843	425.275 ± 45.551	358.533 ± 79.452	
Week 8	439.600 ± 100.475	423.671 ± 78.068	468.713 ± 46.923	405.611 ± 87.332	
Week 9	481.667 ± 101.833	460.900 ± 74.099	514.175 ± 59.457	445.367 ± 97.409	
Week 10	520.856 ± 96.703	499.129 ± 75.396	555.300 ± 60.395	485.744 ± 97.134	
Week 11	559.978 ± 96.092	527.500 ± 78.077	598.975 ± 55.811	520.022 ± 105.205	
Week 12	597.211 ± 99.830	563.000 ± 62.048	630.525 ± 51.870	558.178 ± 115.221	
Week 13	633.633 ± 97.092	593.414 ± 61.852	661.375 ± 49.914	581.011 ± 113.495	
Week 14	675.367 ± 103.378	630.471 ± 52.093	695.963 ± 48.362	619.433 ± 114.489	
Week 15	699.567 ± 103.453	652.729 ± 57.468	722.275 ± 51.114	649.522 ± 114.125	
Week 16	725.978 ± 104.559	672.657 ± 57.643	752.175 ± 59.384	674.000 ± 113.894	

Table S10. Relative organ weights at four months of age (raw means and SD)

**Sex**	**Maternal diet**	Brain wgt	Liver wgt	Heart wgt	Kidney wgt	Adrenal wgt	Testis wgt	Subcut. wgt	Visc. wgt
**Male**	**Optimal (*n* = 8)**	0.4945 ± 0.06404	3.055 ± 0.3289	0.2562 ± 0.008244	0.2943 ± 0.03395	0.01884 ± 0.004228	0.2422 ± 0.04713	0.7671 ± 0.2709	0.4775 ± 0.1721
	**Low (*n* = 7)**	0.5096 ± 0.04471	2.947 ± 0.2975	0.2689 ± 0.01156	0.3013 ± 0.02089	0.02134 ± 0.003771	0.1597 ± 0.1441	0.9128 ± 0.2227	0.5324 ± 0.09271

	**Low-optimal (*n* = 8)**	0.5159 ± 0.04648	2.911 ± 0.3213	0.2524 ± 0.02500	0.3030 ± 0.03292	0.01974 ± 0.003393	0.2166 ± 0.03725	0.8575 ± 0.1343	0.6036 ± 0.1113
	**Optimal-low (*n* = 8)**	0.5761 ± 0.03415	3.210 ± 0.2334	0.2593 ± 0.01067	0.3066 ± 0.02681	0.01874 ± 0.002752	0.2377 ± 0.03843	0.5787 ± 0.1122	0.4250 ± 0.08357
**Model p-value (male)**		0.0130*	0.2485	0.2313	0.8639	0.4877	0.1781	0.0317*	0.0395*
**Female**	**Optimal (*n* = 9)**	0.5332 ± 0.08175	3.298 ± 0.4039	0.2605 ± 0.02060	0.2777 ± 0.02865	0.02461 ± 0.003530	-	1.031 ± 0.2962	0.5862 ± 0.1804
	**Low (*n* = 7)**	0.5569 ± 0.07053	3.230 ± 0.4603	0.2745 ± 0.02581	0.3038 ± 0.03091	0.02109 ± 0.001920	-	1.217 ± 0.4429	0.5305 ± 0.1704
	**Low-optimal (*n* = 8)**	0.5376 ± 0.05055	3.347 ± 0.2574	0.2647 ± 0.01764	0.3014 ± 0.03347	0.02379 ± 0.001950	-	1.250 ± 0.1887	0.7257 ± 0.1157
	**Optimal-low (*n* = 9)**	0.5736 ± 0.07682	3.353 ± 0.5209	0.2698 ± 0.01746	0.3153 ± 0.01701	0.02567 ± 0.004446	-	1.195 ± 0.1576	0.5899 ± 0.1240
**Model p-value (female)**		0.6248	0.9379	0.5700	0.0528	0.0590	-	0.4195	0.0636

All organ weights are in grams and are expressed as a percentage of body weight (g) on the day of euthanasia.

Table S11. Oral glucose tolerance tests (raw means and SD)

**Sex**	**Maternal diet**	0 min (baseline)	30 min	60 min	120 min	180 min
**Male**	**Optimal (*n* = 8)**	8.150 ± 1.434	13.475 ± 3.415	14.238 ± 4.039	8.900 ± 2.746	7.338 ± 1.965
	**Low (*n* = 6)**	8.233 ± 1.309	14.200 ± 2.742	14.400 ± 1.161	9.900 ± 2.005	8.233 ± 1.762
	**Low-optimal (*n* = 5)**	7.933 ± 1.150	15.383 ± 1.895	17.600 ± 2.351	12.717 ± 2.361	7.717 ± 2.028
	**Optimal-low (*n* = 8)**	7.880 ± 0.701	13.880 ± 3.210	15.820 ± 2.787	10.940 ± 2.360	6.875 ± 0.936
**Model p-value (male)**		0.9524	0.6767	0.1742	0.0526*	0.7770
**Female**	**Optimal (*n* = 8)**	8.188 ± 0.728	12.650 ± 2.089	11.850 ± 2.512	7.443 ± 1.715	6.667 ± 1.973
	**Low (*n* = 8)**	7.538 ± 0.935	13.663 ± 1.544	14.100 ± 2.613	8.925 ± 3.023	6.267 ± 0.814
	**Low-optimal (*n* = 5)**	9.025 ± 0.591	15.625 ± 2.300	15.375 ± 3.420	10.625 ± 2.737	8.750 ± 0.636
	**Optimal-low (*n* = 9)**	8.767 ± 0.656	14.556 ± 2.621	14.022 ± 3.168	9.011 ± 1.269	7.800 ± 0.497
**Model p-value (female)**		0.0067*	0.1360	0.1701	0.1358	0.1336

Table S12. Offspring salivary cortisol concentrations (raw means and SD)

**Sex**	**Maternal diet**	Day 0	Day 7	Day 25	d0 vs. d7 p-value	d0 vs. d25 p-value	d7 vs. d25 p-value
**Male**	**Optimal**	52.20 ± 32.33 (*n* = 20)	14.81 ± 10.09 (*n* = 12)	16.62 ± 12.91 (*n* = 14)	0.0001*	0.0008*	>0.9999
	**Low**	63.66 ± 37.60 (*n* = 20)	8.507 ± 4.713 (*n* = 12)	14.87 ± 9.008 (*n* = 14)	<0.0001*	<0.0008*	0.8940
	**Low-optimal**	52.62 ± 28.65 (*n* = 8)	10.67 ± 6.478 (*n* = 10)	10.55 ± 4.235 (*n* = 10)	0.0003*	0.0079*	0.3406
	**Optimal-low**	70.69 ± 25.16 (*n* = 8)	22.84 ± 18.01 (*n* = 8)	9.917 ± 6.520 (*n* = 10)	0.0032*	0.0007*	>0.9999
**Model p-value (male)**		0.2883	0.2274	0.4473	-	-	-
**Female**	**Optimal**	62.84 ± 43.75 (*n* = 20)	12.20 ± 12.77 (*n* = 12)	17.63 ± 11.35 (*n* = 14)	<0.0001*	0.0041*	>0.9999

	**Low**	51.34 ± 28.05 (*n* = 20)	19.98 ± 17.87 (*n* = 12)	19.17 ± 17.30 (*n* = 14)	0.0026*	0.0029*	>0.9999
	**Low-optimal**	71.23 ± 51.86 (*n* = 8)	7.764 ± 4.534 (*n* = 10)	13.17 ± 11.35 (*n* = 10)	0.0003*	0.0073*	>0.9999
	**Optimal-low**	66.96 ± 20.19 (*n* = 8)	10.14 ± 5.549 (*n* = 8)	19.18 ± 14.20 (*n* = 10)	<0.0001*	<0.0001*	0.4052
**Model p-value (female)**		0.4145	0.1466	0.5995	-	-	-
